# Prospective observations study protocol to investigate cost-effectiveness of various prenatal test strategies after the introduction of noninvasive prenatal testing

**DOI:** 10.1186/s12884-018-1930-y

**Published:** 2018-07-24

**Authors:** So Yeon Kim, Seung Mi Lee, Jong Kwan Jun, You Jung Han, Min Hyoung Kim, Jae-Yoon Shim, Mi-Young Lee, Soo-young Oh, JoonHo Lee, Soo Hyun Kim, Dong Hyun Cha, Geum Joon Cho, Han-Sung Kwon, Byoung Jae Kim, Mi Hye Park, Hee Young Cho, Hyun Sun Ko, Jeonghoon Ahn, Hyun Mee Ryu

**Affiliations:** 1 Department of Obstetrics and Gynecology, Seoul National University College of Medicine, Seoul, Republic of Korea; 2 Department of Obstetrics and Gynecology, Cheil General Hospital and Women’s Healthcare Center, Dankook University College of Medicine, Seoul, Republic of Korea; 3 Department of Obstetrics and Gynecology, University of Ulsan College of Medicine, Asan Medical Center, Seoul, Republic of Korea; 4 Department of Obstetrics and Gynecology, Samsung Medical Center, Sungkyunkwan University School of Medicine, Seoul, Republic of Korea; 5 Department of Obstetrics and Gynecology, Institute of Women’s Life Medical Science, Yonsei University College of Medicine, Yonsei University Health System, Seoul, Republic of Korea; 6 Department of Obstetrics and Gynecology, CHA Gangnam Medical Center, CHA University, Seoul, Republic of Korea; 7 Department of Obstetrics and Gynecology, Korea University College of Medicine, Seoul, Republic of Korea; 8 Department of Obstetrics and Gynecology, Konkuk University School of Medicine, Seoul, Republic of Korea; 9 Department of Obstetrics and Gynecology, Seoul Metropolitan Government Seoul National University Boramae Medical Center, Seoul, Republic of Korea; 10 Department of Obstetrics and Gynecology, Ewha Womans University, Seoul, Republic of Korea; 11 Department of Obstetrics and Gynecology, Bundang CHA Medical Center, CHA University School of Medicine, Seongnam, Republic of Korea; 12 Department of Obstetrics and Gynecology, Catholic University of Korea College of Medicine, Seoul, Republic of Korea; 13 Department of Health Convergence, Ewha Womans University, Seoul, Republic of Korea

**Keywords:** Prenatal diagnosis, NIPT, Prenatal screening test, Prenatal genetic counseling, Clinical practice guidelines

## Abstract

**Background:**

Among the non-invasive screening methods for the identification of fetal aneuploidy, NIPT (non-invasive prenatal testing) shows the highest sensitivity and specificity in high-risk pregnancies. Due to the low false positive rate of NIPT, it is assumed that the implementation of NIPT as a primary screening method may reduce the number of invasive fetal tests and result in a similar or lowered cost in the overall detection of Down syndrome. However, most previous studies are based on theoretical economic analysis. This study aims to determine the cost effectiveness of various prenatal test strategies, including NIPT, in real clinical settings in both low risk and high risk pregnancies.

**Methods/design:**

In this prospective observational study, women (< 24 weeks) with singleton or twin pregnancies will be enrolled in 12 different healthcare institutions. The participants will be grouped based on the risks of fetal chromosomal abnormalities and will be counseled on the various screening or diagnostic methods, including NIPT, according to the aneuploidy risk. The final decision on screening or diagnostic methods will be made by patients after counseling. Questionnaires regarding factors affecting the decision on prenatal test will be answered by the participants and physicians. The economic analysis on final total costs will be compared according to the various prenatal test strategies.

**Discussion:**

The results of present study are expected to have a significant impact on national policies in determining Korean prenatal screening test strategies and to help in developing novel and effective prenatal screening tests in the future.

**Electronic supplementary material:**

The online version of this article (10.1186/s12884-018-1930-y) contains supplementary material, which is available to authorized users.

## Background

Aneuploidy is defined as a numerical abnormality of chromosomes and is reported to have an incidence of 1 out of 160 live births [1]. Prenatal diagnosis of aneuploidy is crucial, since it is associated with an increased perinatal morbidity and mortality. In addition, the presence of fetal aneuploidy can affect decision making in fetal treatment, screening, and method of delivery.

Several strategies are currently available for the identification of fetal aneuploidy [2]. Invasive test includes chorionic villi sampling, amniocentesis, and cordocentesis, and is usually indicated for high-risk pregnant women in order to diagnose aneuploidy. The procedure-related risk of this invasive test amounts to 1/300–1/500 within two weeks [3, 4], which restricts this tests from being universally used in low-risk pregnancies. In addition, such an invasive test requires highly trained professionals for the diagnostic procedure. The conventional non-invasive screening tests include maternal serum screening with biochemical markers or ultrasound examination, such as the measurement of fetal nuchal translucency (NT). Among the non-invasive screening methods, triple test and quad test have the accuracy rate of 61–70% and 74–81%, respectively in diagnosing Down syndrome, with the false positive rate of 5% [2, 5]. At 12-week pregnancy, using maternal blood markers, namely, PAPP-A and free beta-hCG, has only 79–87% accuracy rate, with 5% false positive rate [5]. Recently, integrated test involving maternal serum test combined with sonographic results through the first to mid-trimester pregnancy has been introduced, with limited detection rate of 94–96% [5].

Another method introduced in the USA in 2011 and now widely used in 60 different countries is analyzing fetal cell-free DNA in maternal blood sample which is called as NIPT (non-invasive prenatal testing). In recent guidelines, the NIPT is indicated mainly in high-risk pregnant women [6] and shows the highest sensitivity (> 99%) with low false positive rate (<= 0.15%) for the detection of Down syndrome in high-risk pregnancies [7–12].

Due to this low false positive rate, it is assumed that the implementation of NIPT as a screening method may reduce the number of invasive fetal tests. In the study of Larion et al., NIPT’s high detection rate reduces undue invasive prenatal screening tests [13], therefore reducing fetal mortality rate caused by conventional invasive tests.

In terms of cost effectiveness, the decreased number of invasive tests may result in a similar or lowered cost in the overall detection of Down syndrome, as compared to other screening or diagnostic methods. NIPT is expensive, with the associated cost ranging from $795 to $2900 depending on the country; However, the high accuracy rate of NIPT has a value in reducing invasive prenatal tests [1, 13]. These conflicting points (high cost and a value in reduction of invasive test) raise the questions about the cost effectiveness of NIPT in both high-risk and low-risk pregnant women. However, the advantage may be more critical in high-risk pregnancies, although clinical application of NIPT to all pregnant women may not be economically practical.

In the literature, there have been several studies on the cost effectiveness of NIPT (Table 1). Most studies are based on theoretical economic analysis, especially in high-risk pregnancies [14–17]. In England, prospective observational study has been conducted and showed that NIPT decreased invasive tests with same detection rate [18]. This England study was the first study on the effectiveness of NIPT in real world situation than in theoretical model, but this study did not included low risk women (included women with aneuploidy risk of at least 1/1000).

**Table 1:** Several studies for determining cost effectiveness of NIPT

Study	Method of analysis	Study population	Prenatal tests strategies	Result
Alice C. AYRES et al., 2014 [14]	Decision-analytic model	General population	Current practice and NIPT	Most cost-effective for women over 40 years of age
Anjali J. Kaimal et al., 2015 [15]	Decision-analytic model	General population	Chromosomal microarray, miltiple marker screening, cell-free DNA screening, NT screening alone, in combination, or in sequence	NIPT is the most cost-effective after primary screen method at age 40 years and older
Genevieve Fairbrother et al., 2015 [16]	Decision-analytic model	General population	NIPT, first trimester combined screening (FTS)	NIPT is more economical, below $453
Brandon S. Walker et al., 2015 [17]	Decision-analytic model	General population	Contingent NIPT, conventional maternal serum screening (MSS), universal NIPT	Universal NIPT is more cost-effective from a societal perspective view
Lyn S Chitty et al., 2016 [18]	Real clinical setting	Pregnant women with risk for Down syndrome of at least 1/1000	Contingent NIPT, Down syndrome screening program (DSS)	NIPT as a contingent test within DSS program can make more effective outcome of prenatal care
*Current study*	*Real clinical setting*	*General population*	*Maternal serum test (dual test, triple test, quad test, integrated test, sequential test, and contingent test), invasive test (CVS, amniocentesis, cordocentesis), NIPT*	

To determine this issue, we aimed to evaluate the cost effectiveness of different prenatal test strategies for the identification of fetal aneuploidy, including NIPT, in real clinical setting in both low risk and high risk pregnancies.

## Methods/design

### Study design

**Fig. 1:**
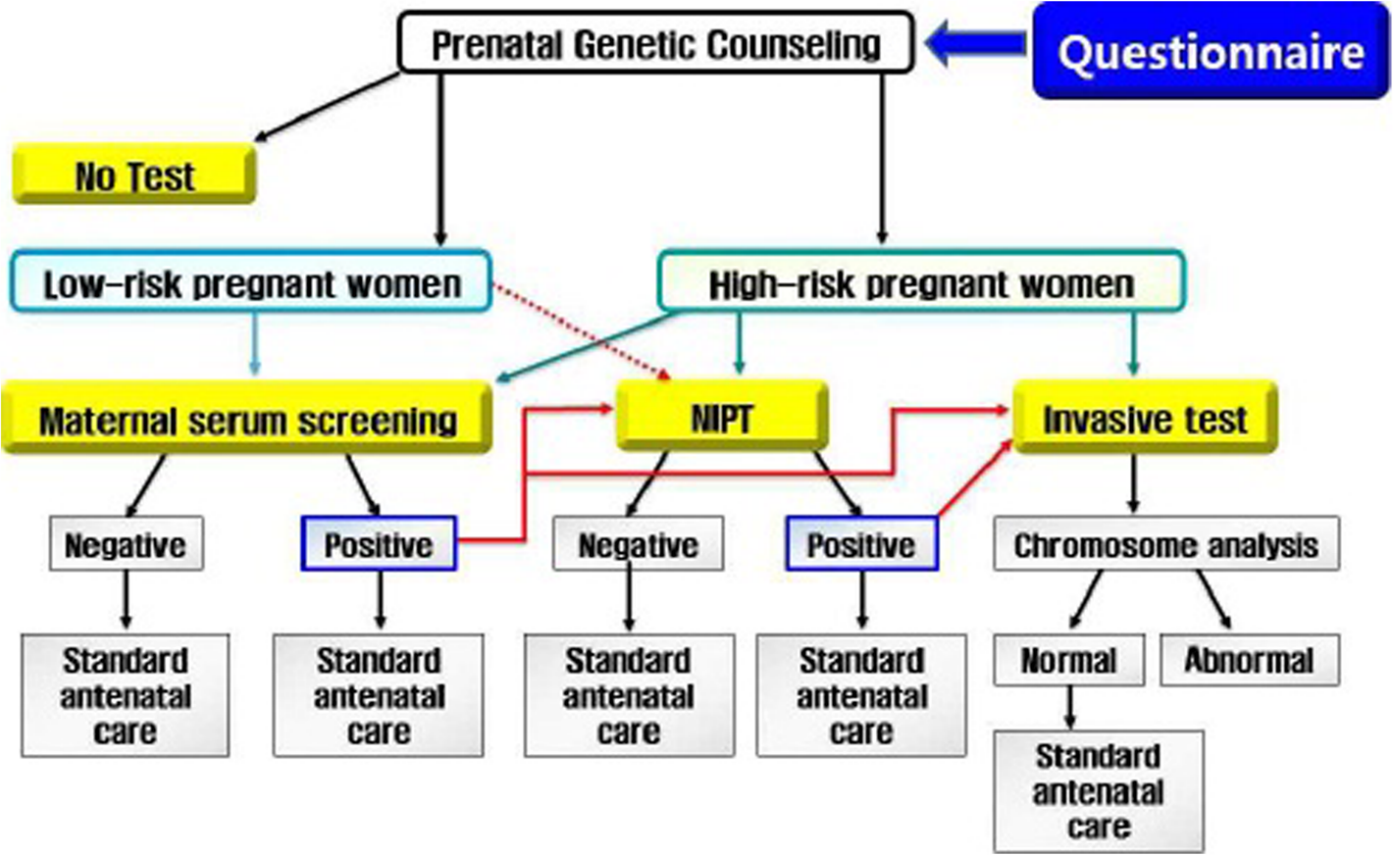
Protocol diagram

This is a multi-center prospective observational study. Fig. 1 depicts the study design with a clinical pathway. The participants will be counseled on various screening or diagnostic methods, based on risks of fetal chromosomal abnormalities. The final decision on screening or diagnostic methods will be made by patients after counseling.

### Population and recruitment

Singleton or twin pregnant women (< 24 weeks of gestation) who are candidates for the identification of fetal aneuploidy will be enrolled in 12 different healthcare institutions between June 2016 and October 2018. The participating hospitals include teaching hospitals in urban areas.

### Sample size

A prior sample size calculation was performed to determine how many participants would be needed to estimate sensitivity of the NIPT. We estimated that the prevalence of Down syndrome would be 3% and the proportion of NIPT tests among prenatal screening tests would be 15% in high-risk pregnancies from the participating health centers, in which the frequency of high risk-pregnancies was estimated to amount to 33% (1/3) of the total number of pregnancies. With the predetermined sensitivity of 99% which is ascertained by previous studies and marginal error of 0.07, 259 women who choose NIPT among prenatal screening tests and a total of 5174 pregnant women would be required. Accounting for the dropout rate, we estimated the minimum sample size as 6000 participants. The 12 participating health care centers had a total of about 17,000 births and about 11,000 maternal serum screenings in one year.

### Inclusion criteria

Inclusion criteria are as follows: (1) singleton/twin pregnant women who present at participating institutions before 24 weeks gestation and (2) candidates for the identification of fetal aneuploidy; (3) 18 years of age or older; (4) Asian ethnicity; (5) ability to read in and speak Korean; (6) provided written informed consent.

### Exclusion criteria

Exclusion criteria are as follows: (1) higher-order multifetal pregnancy; (2) women who visit after 24 weeks; (3) younger than 18 years of age; (4) ethnic background other than Asian.

### Classification of fetal aneuploidy risk

The participants will be grouped based on the risks of fetal chromosomal abnormalities.

Group 1: low risk (no specific risk).

Group 2: advanced maternal age, 35 years or older at the time of delivery.

Group 3: Positive result in maternal serum biochemical test.

Group 4: Abnormal ultrasonographic findings associated with increased risk of aneuploidy.

4–1: increased nuchal translucency (NT).

4–2: presence of soft marker – ultrasonographic findings that are not major anomalies, but indicative of increased risks of chromosomal anomalies (brachycephaly or shortened frontal lobe, clinodactyly, echogenic bowel, flat facies, echogenic intracardiac foci, nasal bone absence or hypoplasia, nuchal fold thickening, pyelectasia, sandal gap between the 1st and 2nd toes, shortened ear length, single transverse palmar crease, single umbilical artery, short long bone, or widened iliac angle).

4–3: major anomaly.

Group 5: prior pregnancy history.

5–1: chromosomal abnormality.

5–1-a: trisomy 13, 18, or 21.

5–1-b: other chromosomal abnormality.

(ex. Tuner syndrome, Robertsonian translocations, etc.)

5–2: congenital anomaly.

Group 6: Maternal or paternal chromosomal abnormality.

6–1: increased risk of trisomy 21, 13.

6–2: other chromosomal abnormality.

Group 7: Positive result in NIPT.

Group 8: Genetic counselling due to abnormal results of invasive test.

### Counseling and decision on prenatal test

The participants will be counseled on the various prenatal tests, including maternal serum screening, NIPT, and invasive prenatal test. The available maternal serum screening tests in Korea include dual test, triple test, quad test, integrated test, sequential test, and contingent test. Available diagnostic tests include chorionic villi sampling, amniocentesis, and cordocentesis.

For women classified as high-risk group on maternal serum test (group 3) will be recommended to NIPT or invasive test. And invasive test will be recommended for women with positive result in NIPT (groups 7), or with markedly increased NT or with major anomaly (groups 4–1, 4–3). The following tests are the firstly recommended tests by a physician, based on the classification of fetal aneuploidy risk.

Group 1: Maternal serum screening test, NIPT.

Group 2: Maternal serum screening test, NIPT, Invasive test.

Group 3: NIPT, Invasive test.

Group 4–1, 4–3: Invasive test.

Group 4–2: Maternal serum screening test, NIPT, invasive test.

Group 5–1: NIPT, Invasive test.

Group 5–2: Maternal serum screening test, NIPT, invasive test.

Group 6–1: NIPT, Invasive test.

Group 6–2: Invasive test.

Group 7: Invasive test.

However, the final decision on screening or diagnostic methods will be made by patients after counseling, in the awareness of their costs.

### Maternal serum screening test

Maternal serum screening test includes double marker (PAPP-A and free beta-hCG), Triple marker (AFP, total hCG, uE3), Quad marker (AFP, total hCG, uE3, Inhibin A), integrated test, sequential test, and contingent test. In the integrated test, the risk will be tested by the combination of the first trimester markers (serum PAPP-A and NT in ultrasound) and the second trimester markers (AFP, total hCG, uE3, Inhibin A). For sequential test, risk will be calculated separately in the first trimester (free-hCG, PAPP-A and NT) and in the second trimester (quad test). Each test will be performed in individual hospitals or by commercial services.

### Invasive test (Cytogenic test)

Cytogenic analysis consists of invasive tests, such as chorionic villous sampling, amniocentesis, and cordocentesis. The final results of these tests will be confirmed by conventional karyotyping. After two weeks, the complications associated with procedures will be determined by the presence of preterm premature rupture of membranes, preterm labor, fetal death in utero, and other complications.

### Fetal ultrasound test

In the first trimester, fetal nuchal translucency will be measured according to the nuchal translucency measurement protocol by Fetal Medicine Foundation. In the 2nd trimester (usually after 18 weeks of gestation), level II ultrasound will be performed for the detection of fetal anomalies.

### NIPT

The level of risk on Down syndrome, Edward syndrome, Patau syndrome, sex chromosomal abnormality will be analyzed in pregnant women who choose NIPT. The NIPT will be performed by several commercially available tests in Korea.

### Investigation of the factors affecting the decision

Factors affecting the decision on the prenatal test will be assessed with the questionnaire answered by the participants (Additional file 1) and physicians (Additional file 2). The patients' questionnaire will be developed with the modification of that used in the study previously published by Lewis C. et al. (PMID: 24433394) In addition, participants will be asked about demographic information, past medical history, and obstetrical history, including history of major anomaly, genetic disorder, or aneuploidy in prior pregnancy.

### Delivery outcome

The data on the result of prenatal tests and pregnancy/neonatal outcomes will be gathered in a subsequent review of medical records. The information on gestational age at delivery, neonatal birth weights, the presence of congenital anomalies, and postnatal cytogenetic study (if performed) will be collected.

### The cost-effective analysis

A cost-utility analysis comparing various prenatal test strategies will be performed from the societal perspective. Transition probabilities and cost data will be estimated from the clinical data collected from 12 participating health centers. Contingent valuation scenarios will be used to solicit utility data for major health states, such as aneuploidy and miscarriage among the pregnant women who have agreed to participate in the study. The incremental cost effectiveness ratio (ICER) analysis will be used to identify the most cost-effective strategy.

## Discussion

In this study, we will try to find out which method is more cost-effective for detection of fetal aneuploidy, including NIPT.

NIPT is expensive ranging from $795 to 2900 depending on countries in the literature, and the NIPT cost in Korea is between 550,000 and 800,000 KRW. However, the high accuracy rate of NIPT has a value in reducing invasive prenatal tests [1, 13]. These conflicting points (high cost and a value in reduction of invasive test) raised the questions on the cost-effectiveness of NIPT in both high-risk and low-risk pregnant women. In the literature, there have been several studies on the cost-effectiveness of NIPT (Table 1). The NIPT has been shown to be cost-effective, especially in high risk patients. However, the cost-effectiveness of NIPT in low risk women has not been well determined. In addition, these previous studies are mostly based on theoretical economic analysis and clinical data in a ‘real-world situation’ has not been well evaluated. Lastly, the cost-effectiveness analysis in Korean population seems to be important, because the mean age of Korean pregnant women was higher than other countries (such as USA and England) and old age itself can be a high risk factor [19].

To our knowledge, this study will be the first study which evaluated the cost effectiveness of NIPT in low risk pregnancies in reality. In addition, this will be the first study determining the impact of NIPT in twin pregnancies.

The results of this study are expected to have a significant impact on improving national health, and related policies (Table 2). First, on national health level, prenatal screening guidelines would reduce unnecessary invasive tests and result related abortion. Secondly, prenatal screening costs would be reduced. Third, more effective government policies would be made for prenatal care and gathering evidence for improving low birth rates. Finally, this would be basis of effective prenatal screening.

**Table 2:** Subsequent studies using the data derived from our study

Cost-effectiveness study	Cost-effectiveness of various prenatal tests in real clinical setting - In both low risk and high risk pregnancy - In both singleton and twin pregnancy
Factors affecting the decision	1) Questionnaire Study: factors affecting the decision on prenatal test by both participants, husbands and attending physicians in real clinical setting 2) Clinical factors affecting the decision in real clinical setting - In both low risk and high risk pregnancy - In both singleton and twin pregnancy
NIPT	1) Clinical aspect: Introduction of NIPT and its effects on selection of prenatal test including invasive diagnostic tests - In both low risk and high risk pregnancy - In both singleton and twin pregnancy 2) Diagnostic aspect: the diagnostic accuracy and no-call reports of NIPT - In both low risk and high risk pregnancy - In both singleton and twin pregnancy 3) The usefulness of NIPT in structural malformation or in increased nuchal translucency 4) The risk of major structural malformation according to the results of various prenatal tests
Maternal serum screening	Evaluation of adverse pregnancy outcomes according to the maternal serum screening test - In both low risk and high risk pregnancy - In both singleton and twin pregnancy
Invasive test study	1) Complication rate including abortion in invasive prenatal tests in Korean clinical settings - In both low risk and high risk pregnancy - In both singleton and twin pregnancy 2) Change in abortion rate after the introduction of NIPT
Guidelines	1) Development of guidelines for prenatal screening test 2) Development of guidelines for prenatal diagnostic test

## Additional files

Additional file 1: Patients Questionnaire: Korean version and Patients Quesionnaire: English version. (ZIP 524 kb)

Additional file 2: Physicians Questionnaire: Korean version and Physicians Questionnaire: English version. (ZIP 536 kb)

## Abbreviations

AFP: alpha-fetoprotein
hCG: human chorionic gonadotropin
KRW: South Korean won
NIPT: non-invasive prenatal testing
NT: nuchal translucency
PAPP-A: pregnancy-associated plasma protein A
uE3: unconjugated estriol
