# Supplementary material for: Prospective observations study protocol to investigate cost-effectiveness of various prenatal test strategies after the introduction of noninvasive prenatal testing
Source: BMC Pregnancy Childbirth. 2018 Jul 24;18:307. doi: 10.1186/s12884-018-1930-y (PMC6056912; doi:10.1186/s12884-018-1930-y)
Supplement: Supplementary file 1 — Patients Questionnaire: Korean version and Patients Quesionnaire: English version. (ZIP 524 kb) [file 12884_2018_1930_MOESM1_ESM.zip › (Additional file 1) patients questionnaire_English versionR2.pdf]

Serial Number: ________

Questionnaire for an Awareness-Survey on Antenatal tests for Down syndrome

The information you provide in this questionnaire will be used for our survey on the awareness of expecting mothers and their spouses on various tests to screen fetuses affected by Down syndrome.

First of all, please read the following text, which is provided to brief you into the various antenatal tests for Down syndrome.

Introduction to Antenatal testing for Down Syndrome

Normally, a person has 46 chromosomes. These are organized in 22 pairs, from No. 1 to No. 22, and a pair of sex chromosomes.

Down syndrome is one of the most common genetic disorders, in which an extra full or partial copy of No. 21 chromosome 21 exists.

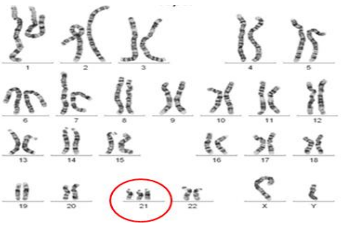

To detect fetuses affected by Down syndrome during pregnancy, pregnant women will be tested for Down syndrome from their 1st trimester (after 10th week of gestational age).

- Normally, antenatal tests for Down syndrome consist of **screening tests** and **confirmative diagnostic tests**.

-Firstly, there are several kinds of **screening tests** for Down syndrome. In general screening tests **combining the maternal blood with ultrasonographic examination** are commonly used. The detection rate of these screening tests for Down syndrome varies according to the methods of examination and is known to be approximately **65-95%.**

- Diagnostic tests include (1) the **chorionic villus sampling** that is performed between 10 and 13 weeks of gestational age, (2) **amniocentesis** after 15 weeks of gestational age, and (3) the **cordocentesis**, which is performed usually after 20 weeks of gestational age. However, these diagnostic tests also have **the risk of the procedure-related miscarriage in 0.1 ~ 0.3%**. Therefore, a step-wise approach with screening and diagnostic tests is performed.

- With a recent break-through, we now have another option for the screening test, where the **fetal DNA** in the maternal blood (cell-free fetal DNA) is tested to detect Down syndrome with **98% accuracy**.

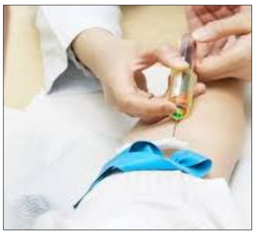

Maternal serum test

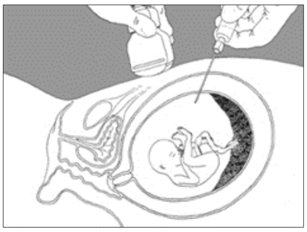

Amniocentesis

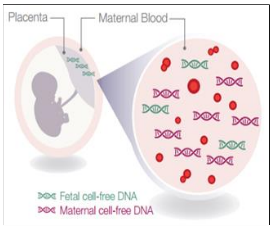

Fetal DNA Test

Here are the questions for you. Consider you, or your wife is pregnant now.

1. Are you willing to undergo antenatal testing for Down syndrome during this pregnancy?
   ① Yes ☞ Proceed to Question No. 2 on Page 3.
   ② No ☞ Proceed to Question No. 5 on Page 4.

2. The following statements are the factors that could have affected your decision to **get antenatal testing for Down syndrome**. How important was each of them?

	Not at all important.	Not so much important.	Moderate	Somewhat important.	Extremely important
Because I am willing to make plans and maintain pregnancy, so that we will raise the baby, even if he or she has Down syndrome.	1	2	3	4	5
Because I think it is practically impossible for me, my spouse, or other family members to raise a baby with Down syndrome.	1	2	3	4	5
Because I want to get as much information as I can get about the fetus.	1	2	3	4	5
Because my spouse or family members want me to take the test.	1	2	3	4	5
Because all other expecting mothers are receiving the tests.	1	2	3	4	5
Because the OBGY doctor (health professional) recommends the test.	1	2	3	4	5
Because I think I need to take as many tests as I can during my pregnancy.	1	2	3	4	5
Others: ______					

3. Who is the person that made the **final decision about antenatal testing for Down syndrome**? (Pick only one answer)

① Me
② My spouse
③ A joint decision between me and my spouse
④ Family members (other than me and my spouse)
⑤ OBGY doctor (health professional)
⑥ Others: ______

4. As we discussed earlier, there are two approaches to detect Down syndrome, either (1) **taking the screening tests first before getting a diagnostic test**, or (2) **skipping the screening tests and getting a diagnostic test right away**. Which approach do you prefer?

① Take the screening tests first and proceed to diagnostic tests ☞ Proceed to Scenario 1 in Page
② Skip the screening and the diagnostic tests right away. ☞ Proceed to Scenario 2 in Page 9.

5. The following statements are the factors that could have affected your decision **not to get antenatal testing for Down syndrome**. How important was each of them?

	Not at all important.	Not so much important.	Moderate	Somewhat important.	Extremely important
Because I am having the baby regardless of the test results.	1	2	3	4	5
Because I think it is possible for me, my spouse, or other family members to raise a baby with Down's syndrome.	1	2	3	4	5
Because I do not want to get an invasive test that can be dangerous to the unborn child.	1	2	3	4	5
Because I am so scared and worried of getting a high risk result for Down's syndrome and would rather not get the tests at all.	1	2	3	4	5
Because I do not want to know too much about the baby before he or she is born.	1	2	3	4	5
My spouse or family members do not want it.	1	2	3	4	5
Because I do not want it.	1	2	3	4	5
Because the test is too expensive.	1	2	3	4	5
Because it is still illegal to terminate pregnancy even if I discover Down's syndrome through the test.	1	2	3	4	5
Others: ______________________________					

6. Who is the person that made the **final decision not to get antenatal tests for Down syndrome**? (Pick only one answer)

① Me
② My spouse
③ A joint decision between me and my spouse
④ Family members (other than me and my spouse)
⑤ OBGY doctor (health professional)
⑥ Others: ______________________________

☞ Now, proceed to Scenario 1 in Page 5.

Scenario 1: Screening test for Down syndrome Using maternal blood samples and ultrasonographic examination.

Recently, you found out that you are pregnant. And, you visited an OBGY clinic to find out more about screening tests for Down syndrome. The OBGY doctor explained you about the Down syndrome screening tests as follows:

- The Down syndrome screening tests we are talking about use the **maternal blood samples and ultrasonographic examination**.
- In these tests, 4 or 5 different kinds of proteins in the maternal serum are analyzed in order to detect Down syndrome-affected pregnancy. These tests include **Quad test** and **Integrated test**.
- **Quad test** is performed between 15 to 22 weeks of gestation, while Integrated test can be taken from 11 to 13 weeks of gestation (as the first test) and from 15 to 22 weeks of gestation (as the second test).
- With these tests, it is possible to evaluate the chance of the unborn child you have now being affected by Down's syndrome (for example, as one to 100 or 1 to 10,000, etc.) However, **none of these tests can surely give a confirmatory diagnosis whether your baby has Down syndrome or not.**
- If the test results show a high risk for Down syndrome, it is necessary to proceed to the diagnostic tests to get a confirmatory diagnosis.
- If you are not willing to take these tests using your blood sample and ultrasonographic findings, you may decline to take them.

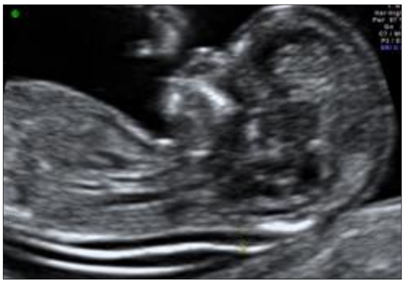

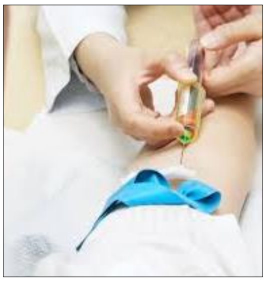

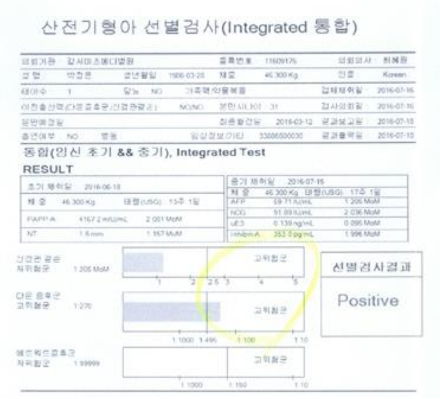

7. When are you going to make your decision whether to receive the **Down syndrome screening tests using the maternal blood samples and ultrasonographic examination**? (Choose only one answer.)

① Right now.

② After thinking about it for several days.

③ I don’t know.

8. Are you willing to receive the above-mentioned **Down syndrome screening tests using the maternal blood samples and ultrasonographic examination**? (Choose only one answer.)

① Definitely yes ☞ Proceed to Question No. 9 on Page 7.

② Probably yes ☞ Proceed to Question No. 9 on Page 7.

③ Probably not ☞ Proceed to Question No. 10 on Page 8.

① Definitely not ☞ Proceed to Question No. 10 on Page 8.

9. If you answer yes to Question No 8, which statement best matches your reason why? (choose **up to three answers**)

Because I am willing to make plans and maintain pregnancy, so that we will raise the baby even if it has Down syndrome.	1
Because I think the test will be helpful for making the decision to terminate the pregnancy or not, as it is practically not possible to raise a child with Down syndrome.	2
Because I want to get as much information as I can get about the fetus.	3
Because the test is completely free of any risks for the baby.	4
Because my spouse or family members want me to take the test.	5
Because all other expecting mothers are receiving the tests.	6
Because the OBGY doctor recommends the test.	7
Because I think I need to take as many tests as I can during my pregnancy.	8
Others: ____________________	

☞ Now, proceed to Scenario 2 in Page 9.

10. If you answered no to Question No 8., which statement best matches your reason why? (Choose **up to three answers**.)

Because I am having the baby regardless of the test results.	1
Because I think it is possible for me, my spouse, or other family members to raise a baby with Down syndrome.	2
Because I do not want to get an invasive test that can be dangerous to the unborn baby.	3
Because I am so scared and worried of getting a high risk result for Down syndrome and would rather not get the tests at all.	4
Because I do not want to know too much about the baby before he or she is born.	5
My spouse or family members do not want it.	6
Because I do not want it.	7
Because the result of this test is a confirmatory diagnosis.	8
Because the test is too expensive.	9
Because it is still illegal to terminate pregnancy even if I discover Down syndrome through the test.	10
Others: ______________________________	

☞ Now, proceed to Scenario 3 on Page 13.

Scenario 2: Diagnostic test for Down syndrome

You decided to take the Down syndrome screening tests using the maternal blood samples and ultrasonographic examination and the results showed that your baby had a high risk for Down syndrome. And, you discussed with your doctor regarding the diagnostic tests for Down syndrome such as **chorionic villus sampling, amniocentesis, and cordocentesis**, and what the doctor told you is the followings;

- The tests mentioned above are the final confirmatory diagnostic tests that **tell you whether your baby actually has Down syndrome or not**. And, the test also indicates **whether there are any structural or quantitative anomalies in the chromosomes of the baby**.
- These tests provide the **highest level of accuracy** as they use the fetal cells.
- For these tests, a needle is punctured through the abdomen of the mother under ultrasonographic guidance and samples of chorionic villi, amniotic fluid or umbilical cord blood are taken. There is a **0.1 to 0.3% possibility of a miscarriage or abortion associated with this test**.
- So, if you do not wish to take these tests, you may skip them.

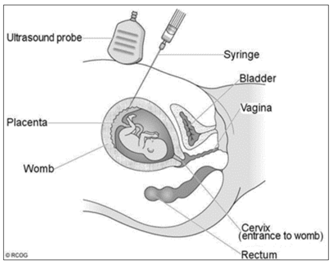

Chorion test

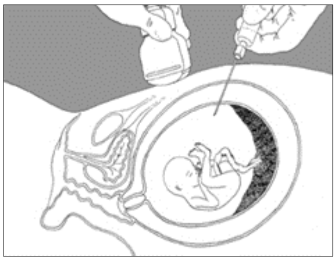

Amniocentesis

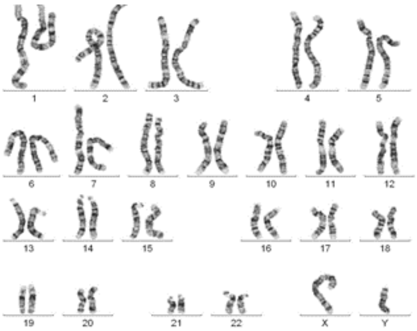

11. When are you going to make your decision whether or not to take the above-mentioned tests? (Choose only one answer.)

① Right now.

② After thinking about it for several days.

③ I don’t know.

12. Are you willing to take the above-mentioned tests? (Choose only one answer.)

① Definitely Yes ☞ Proceed to Question No. 13 on Page 11.

② Probably Yes. ☞ Proceed to Question No. 13 on Page 11.

③ Probably Not. ☞ Proceed to Question No. 14 on Page 12.

① Definitely Not. ☞ Proceed to Question No. 14 on Page 12.

13. If you answered no to Question No 12.., which statement best matches your reason why? (choose **up to three answers.)**

Because I am willing to make plans and maintain pregnancy, so that we will raise the baby, even if he or she has Down syndrome.	1
Because I thought the test would be helpful for making the decision to terminate the pregnancy or not, as it is practically not possible to raise a child with Down syndrome.	2
Because I want to get as much information as I can get about the fetus.	3
Because some of my friends or family members received such diagnostic tests during their pregnancy.	4
Because my spouse or family members want me to take the test.	5
Because all other expecting mothers are receiving the tests.	6
Because the OBGY doctor recommends the test.	7
Because I think I need to take as many tests as I can during my pregnancy.	8
Others: ______________________________	

☞ Now, proceed to Scenario 3 in Page 13.

14. If you answered no to Question No 12., which statement best matches your reason why? (choose **up to three answers.**)

Because there is a risk of miscarriage or abortion.	1
Because I fear the test will be painful and difficult for me to bear.	2
Because I am having the baby regardless of the test results.	3
Because I think it is possible for me, my spouse, or other family members to raise a baby with Down syndrome.	4
Because I am so scared and worried that my child may be diagnosed with Down syndrome and would rather not get the tests at all.	5
Because I do not want to experience a situation where I have to make the decision to terminate the pregnancy or not.	6
Because I do not want to know too much about the baby before he or she is born.	7
My spouse or family members do not want it.	8
Because I do not want it.	9
Because the test is too expensive.	10
Because it is still illegal to terminate a pregnancy even if I discover Down's syndrome through the test.	11
Others: ______________________________	

☞ Now, proceed to Scenario 3 in Page 13.

Scenario 3: Down syndrome screening test using cell-free fetal DNA of maternal blood

We now have a new testing method to detect Down syndrome, which is called the **cell-free fetal DNA test for Down syndrome screening**. As this test method requires only the maternal blood sample, it is called a non-invasive prenatal test (NIPT), which is quite different from the invasive tests such as chorionic villus sampling or amniocentesis. However, in this questionnaire, we will simply refer to them as 'fetal DNA test'.

- The blood of a pregnant woman includes fetal cells and DNAs. And, the fetal DNA test uses **fetal DNA found in the maternal blood to screen Down syndrome.**
- As this test uses the maternal blood, **the risks of miscarriage or abortion are not increased**.
- Also, it has been shown that this method provides more than **98% accuracy in detecting Down syndrome.** The test can be performed as early as from 10 weeks of gestation.
- The fetal DNA test is **not a confirmatory diagnostic test** for Down syndrome. Therefore, even if you have a high-risk result, you **still need to get invasive confirmatory diagnostic tests.**
- In the past, the maternal blood samples had to be sent to laboratories abroad as there were none that could perform such tests in Korea. However, now we have many Korean companies that provide these tests.
- This test is not used to give a confirmatory diagnosis of Down syndrome but its accuracy rate in detecting Down's syndrome is at least more than 98%, and at the same time it is still a non-invasive test without any risks of miscarriage or abortion. Therefore, this test can be performed on patients who are found to be at risk for Down syndrome in other Down syndrome screening tests (Quad test or Integrated test) or the existing Down syndrome screening tests can be replaced with this test.

15. Do you think the fetal DNA test can be useful for pregnant women as a part of the prenatal management program? (Pick only one answer)

① I am sure of it.

② Maybe.

③ Maybe not.

④ Definitely not so.

16. If the fetal DNA test is used as a part of the prenatal management program, are you willing to take the fetal DNA test? (Pick only one answer)

① I am sure of it.

② Maybe.

③ Maybe not.

④ Definitely not so.

17. Theoretically, how do you think the fetal DNA test should be offered? (Pick only one answer)

All pregnant women should receive this test.	1
Only to pregnant women who have been identified as 'high-risk' through the existing screening test.	2
Not only to women identified as high-risk through screening, but also to all women who ask for it.<br>- Expecting mothers who tested as low-risk but still uncertain and worried so that they want to receive the fetal DNA test.<br>- Expecting mothers who proceed to the fetal DNA test without taking existing screening tests, etc.	3
I don't think it should be offered	4
Others: ______________________________	

18. What is the most important factor in your decision whether or not **to receive the cell-free fetal DNA test to screen Down syndrome?** (Pick only one answer)

① Because the test is safe for the fetus. (The risks of miscarriage or abortion are not increased.)

② Because this test lets you know the Down syndrome risk level at a relatively earlier stage of pregnancy, compared to the other screening tests.

③ Because it is more accurate (over 98% accuracy).

④ Because it is easy to take the test. (Only a maternal blood sample is required.)

⑤ I am not going to receive any antenatal tests for Down syndrome.

⑥ Others: ______________________________

☞ Now, proceed to Scenario 4 in Page 16.

Scenario 4: Down syndrome screening test using cell-free fetal DNA of maternal blood

Recently, you found out that you were pregnant. You decided to receive a Down syndrome screening test, which gave a high-risk result. Then, **your doctor explained you about the cell-free fetal DNA test using the fetal DNA found in the maternal blood+**.

- The fetal DNA test is a screening test, but not diagnostic test
- The accuracy level of the cell-free fetal DNA test is **at least 98%**.
- All that is required to take the test is a maternal blood sample, therefore there is **no risk of miscarriage or abortion.**
- As the cell-free fetal DNA test does not give a confirmatory diagnosis, you have to **take a invasive confirmatory diagnostic test, even if your result is a high-risk case.**
- The fetal DNA test is **expensive** costing between 500,000 to 1,000,000 Korean Won.
- You don't have to take the cell-free fetal DNA test if you are not willing to take it.

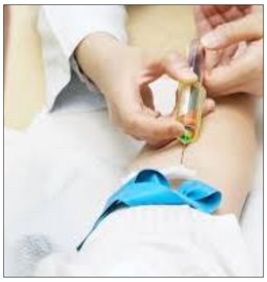

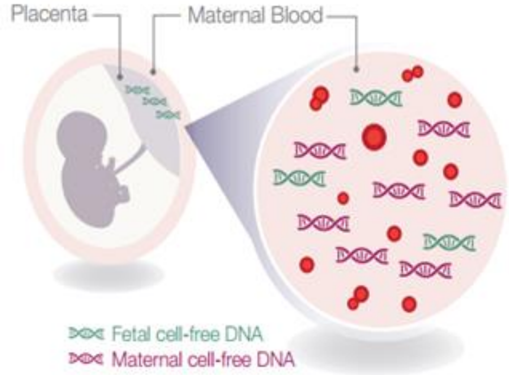

19. When are you going to make your decision whether or not to take the above-mentioned cell-free fetal DNA test? (Choose only one answer.)

① Right now.

② After thinking about it for several days.

③ I don’t know.

20. Are you willing to take the above-mentioned cell-free fetal DNA test? (Choose only one answer.)

① Definitely Yes. ☞ Proceed to Question No. 21 on Page 18.

② Probably Yes. ☞ Proceed to Question No. 21 on Page 18.

③ Probably Not. ☞ Proceed to Question No. 24 on Page 20.

① Definitely Not. ☞ Proceed to Question No. 24 on Page 20.

21. If you answered yes to Question No. 20., which statement best matches your reason why? (choose **up to three answers.**)

Because I am willing to make plans and maintain pregnancy, so that we will raise the baby, even if he or she has Down's syndrome.	1
Because I thought the test would be helpful for making the decision to terminate the pregnancy or not, as it is practically not possible to raise a child with Down's syndrome.	2
Because I want to get as much information as I can get about the fetus.	3
Because the test is completely free of any risks for the fetus.	4
Because my spouse or family members want me to take the test.	5
Because I think other expecting mothers are taking this test, or there are friends or family members who received this test.	6
Because the OBGY doctor recommends the test.	7
Because I think I need to take as many tests as I can during my pregnancy.	8
Others: ______________________________	

22. Will you terminate your pregnancy if the cell-free fetal DNA test tells your child has a chromosome aberration? (Pick only one answer)

① I will terminate the pregnancy immediately.

② I will make the final decision after getting a confirmatory diagnostic test.

③ I don't know.

23. How much are you willing to pay for the cell-free fetal DNA test? (Pick only <u>one</u> answer)

① 50,000 ~ 100,000 KRW

② 100,000 ~ 200,000 KRW

③ 200,000 ~ 400,000 KRW

④ 400,000 ~ 600,000 KRW

⑤ 600,000 ~ 800,000 KRW

⑥ 800,000 ~ 10,00,000 KRW

⑦ > 1 million KRW

⑧ Specify: ____

☞ Thank you. Please proceed to Page 21 and answer the questions regarding yourself.

24. If you answered no to Question No. 20., which statement best matches your reason why? (choose **up to three answers.**)

Because I am having the baby regardless of the test results.	1
Because I think it is possible for me, my spouse, or other family members to raise a baby with Down's syndrome.	2
Because I am so scared and worried of getting a high risk result for Down's syndrome and would rather not get the tests at all.	3
Because I do not want to know too much about the baby before he or she is born.	4
My spouse or family members do not want it	5
Because I do not want it.	6
Because the test result is a confirmatory diagnosis.	7
Because the test is too expensive.	8
Because it is still illegal to terminate pregnancy even if I discover Down's syndrome through the test.	9
Others: ______________________________	

☞ Thank you. Please proceed to Page 21 and answer the questions regarding yourself.

<Personal Information>

25. What is your gender?
   ① Male
   ② Female

26. How old are you? ___

27. What is your highest education qualification?
   ① No qualification.
   ② Elementary school
   ③ Middle school
   ④ High school
   ⑤ Degree level or above
   ⑥ Others: ______________________________

28. Do you have a religion?
   ① None
   ② Catholic
   ③ Protestant
   ④ Buddhism
   ⑤ Islam
   ⑥ Others: ______________________________

29. Are you currently married?
   ① Yes
   ② No

30. Do you have children? (Not counting the current pregnancy)
   ① Yes
   ② No

31. What is the gestational age of current pregnancy? ___ Week

32. Have you ever received any screening tests for Down syndrome using your blood sample and ultrasonographic examination during the current and previous pregnancies?

① Yes

② No

③ I don’t remember.

32-1, If you had received, what was the result?

① High risk

② Low risk

③ I don’t remember.

33. Have you ever received any diagnostic tests for Down syndrome during the current and previous pregnancies?

① Yes

② No

③ I don’t remember.

33-1, If you had received, what was the result?

① Diagnosed with Down syndrome

② Other chromosomal aberration other than Down syndrome

③ Normal result.

④ I don’t remember.

34. Do you have a child with Down’s syndrome?

① Yes

② No

35. Does anyone you know have a child with Down syndrome (including families, relatives, or friends)?

① Yes

② No

<The following question only applies to the persons who answered that they are not willing to receive antenatal tests for Down syndrome in Question No. 2 on Page 2.>

36. You have learned about antenatal tests for Down syndrome from this questionnaire. Are you inclined to receive antenatal tests for Down syndrome test in the future?

① Yes

② No

37. As we discussed earlier, there are two approaches to detect Down syndrome, either (1) taking the screening tests first before getting a diagnostic test, or (2) skipping the screening tests and getting a diagnostic test right away. Which approach do you prefer?

① Take screening tests and then diagnostic tests as necessary.

② Proceed to the diagnostic tests without taking screening tests.

③ I am not willing to take any Down's syndrome tests whatsoever.

38. If the cell-free fetal DNA test is used as a part of the prenatal management program, are you willing to take the cell-free fetal DNA test? (Pick only <u>one</u> answer)

① I am sure of it.

② Maybe.

③ Maybe not.

④ Definitely not so.

⑤ I am not willing to take any Down's syndrome tests whatsoever.

< Thank you very much for your time.>
