# Supplementary material for: Prospective observations study protocol to investigate cost-effectiveness of various prenatal test strategies after the introduction of noninvasive prenatal testing
Source: BMC Pregnancy Childbirth. 2018 Jul 24;18:307. doi: 10.1186/s12884-018-1930-y (PMC6056912; doi:10.1186/s12884-018-1930-y)
Supplement: Supplementary file 1 — Patients Questionnaire: Korean version and Patients Quesionnaire: English version. (ZIP 524 kb) [file 12884_2018_1930_MOESM1_ESM.zip › (Additional file 1) patients questionnaire_korean versionR2.pdf]

일련번호: _______

다운증후군 검사에 대한 인식조사용 설문지

본 설문지는 임신 중에 다운증후군 태아를 발견하기 위한 여러 가지 검사에 대한 임신부와 배우자의 인식을 조사하기 위한 목적으로 사용될 것입니다.

먼저 산전 다운증후군 검사에 대한 이해를 돕기 위해 다음을 읽어보시고 진행하시면 됩니다.

다운증후군 검사 소개

인간의 염색체는 정상적으로 46개입니다. 1번에서 22번 염색체가 각각 2개씩 짝을 지어 존재하며, 성염색체 2개로 구성되어 있습니다.

다운증후군이란 21번 염색체가 2개가 아닌 3개로 이루어진 대표적인 염색체 이상 질환입니다.

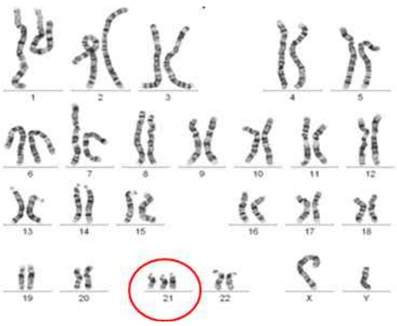

임신 중에 이러한 다운증후군 태아를 발견하기 위해서, 임신부는 임신 제1삼분기 후반(임신 10주 이후)부터 다운증후군 검사를 시행하게 됩니다.

- 다운증후군 검사는 크게 **선별검사**와 **진단검사**로 구분됩니다.

- 먼저, 다운증후군 **선별검사**의 종류는 여러 가지가 있으며, 일반적으로 **임신부의 혈액과 초음파 검사를 이용한 검사**가 널리 사용되고 있습니다. 이러한 선별검사의 다운증후군 태아의 발견율은 검사방법에 따라 다양하며, 대략 **65-95%** 정도로 알려져 있습니다.

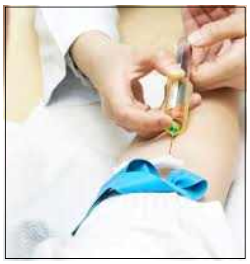

모체혈청검사

- **진단검사**는 ① 임신 10-13주에 시행하는 **융모막검사**, ② 임신 15주 이후에 시행하는 **양수검사**, 그리고 ③ 주로 임신 20주 이후에 시행하는 **제대혈검사**로 나눌 수 있습니다. 그러나, 진단검사는 **시술로 인한 유산 위험율이 0.1-0.3%** 정도로 알려져 있기 때문에, 선별검사와 진단검사를 단계적으로 시행하기도 합니다.

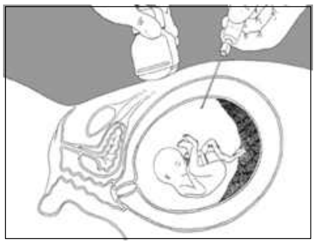

양수검사

- 최근 임신부의 혈액내에 존재하는 **태아DNA**를 이용하여 **98%의 다운증후군 발견율**을 보이는 선별검사가 개발되었습니다.

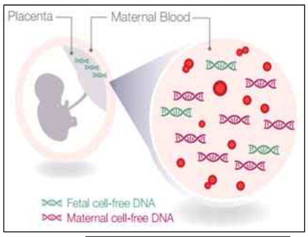

태아 DNA 검사

이제 설문을 시작하시겠습니다. 당신 또는 당신의 아내가 현재 임신 중 입니다.

1. 이번 임신기간에 태아의 다운증후군 검사를 받을 의향이 있나요?

① 예 ☞ 3페이지의 2번 질문으로 넘어가세요.

② 아니오 ☞ 4페이지의 5번 질문으로 넘어가세요.

2. 다음은 **다운증후군 검사를 받기로 결정하는데 영향을 끼친 이유**를 나열하고 있습니다. 각각의 이유가 얼마나 중요하게 작용했습니까?

	전혀 중요하지 않음	별로 중요하지 않음	보통	어느정도 중요	매우 중요
아기가 다운증후군이라 하더라도, 출산 전에 준비하고 계획해서 아기를 낳아서 키울 생각이 있어서	1	2	3	4	5
현실적으로 본인이나 배우자 및 가족들이 다운증후군 아기를 키우는 것이 불가능하다고 생각해서	1	2	3	4	5
태아에 대해서 최대한 많은 정보를 알고 싶어서	1	2	3	4	5
배우자 또는 가족들이 내가 검사하기를 원해서	1	2	3	4	5
다른 임신부들이 모두 검사를 받아서	1	2	3	4	5
산부인과 의사가 권해서	1	2	3	4	5
임신 중 받을 수 있는 모든 검사는 다 받아야 할 것 같아서	1	2	3	4	5
기타: ______________________________					

3. 다운증후군 **검사를 받기로 최종적으로 결정**한 사람은 누구입니까? (**하나**만 선택하세요)

① 본인

② 배우자

③ 본인과 배우자가 상의해서 결정

④ 가족 (본인과 배우자 제외)

⑤ 산부인과 의사

⑥ 기타: ______________________________

4. 다운증후군 검사는 앞서 설명드린대로 ① 선별검사와 진단검사를 **단계적으로 시행**하는 방법과, ② 선별검사를 시행하지 않고 **곧바로 진단검사를 시행**하는 방법이 있습니다. 어떤 검사 방법을 선택하시겠습니까?

① 선별검사와 진단검사를 단계적으로 시행 ☞ 5페이지의 '시나리오 1'로 넘어가세요.

② 선별검사 없이 곧바로 진단검사를 시행 ☞ 9페이지의 '시나리오 2'로 넘어가세요.

5. 다음은 **다운증후군 검사를 받지 않기로 결정하는데 영향을 끼친 이유**를 나열하고 있습니다. 각각의 이유가 얼마나 중요하게 작용했습니까?

	전혀 중요하지 않음	별로 중요하지 않음	보통	어느정도 중요	매우 중요
검사 결과에 상관없이 아기를 낳을 것이어서	1	2	3	4	5
본인이나 배우자 및 가족들이 다운증후군 아기를 키울 수 있다고 생각해서	1	2	3	4	5
아기에게 위험할 가능성이 있는 침습적인 검사는 시행하고 싶지 않아서	1	2	3	4	5
내 아기가 다운증후군의 고위험이라고 결과가 나올까봐 매우 걱정스럽고 불안해서, 차라리 안하는 것이 나을 것 같아서	1	2	3	4	5
분만하기 전까지 태아에 대한 정보를 너무 많이 알고 싶지 않아서	1	2	3	4	5
배우자 또는 가족들이 원치 않아서	1	2	3	4	5
내가 원치 않아서	1	2	3	4	5
검사비가 경제적으로 부담이 되어서	1	2	3	4	5
검사를 시행하여 다운증후군을 발견한다 하더라도 현행 의료법상 다운증후군 태아를 유산을 하는 것은 불법이어서	1	2	3	4	5
기타: ______________________________					

6. 다운증후군 **검사를 받지 않기로 최종적으로 결정**한 사람은 누구입니까? (**하나**만 선택하세요)

① 본인

② 배우자

③ 본인과 배우자가 상의해서 결정

④ 가족 (본인과 배우자 제외)

⑤ 산부인과 의사

⑥ 기타: ______________________________

☞ 이제 5페이지의 '시나리오 1'로 넘어가세요.

시나리오 1: 임신부 혈액과 초음파 검사를 이용한 다운증후군 선별검사

최근에 당신은 임신을 확인하였습니다. 다운증후군 선별검사에 대한 정보를 얻고자 산부인과에 방문하였습니다. 산부인과 의사가 지금부터 시행하려는 다운증후군 선별검사에 대해 다음과 같이 설명하였습니다.

- 지금 설명하고 있는 다운증후군 선별검사는 **임신부의 혈액과 초음파 검사를 이용한 검사**입니다.
- 이 검사는 임신부 혈청내의 4-5가지 단백질을 분석하여 다운증후군을 선별하는 방법으로 **쿼드검사**와 **통합검사** 등이 있습니다.
- **쿼드검사는 임신 15-22주에** 시행하며, **통합검사는 임신 11-13주에 1차 검사, 임신 15-22주에 2차 검사**를 시행합니다.
- 이 검사를 통해 지금 임신한 아기가 다운증후군일 위험도가 높은지 낮은지를 알 수 있습니다 (다운증후군 확률이 1:100 또는 1:10,000 등). 그러나, **지금 시행하려는 선별검사는 현재 임신 중인 태아가 다운증후군인지 아닌지를 확실하게 진단하는 검사는 아닙니다**.
- 이 검사에서 다운증후군 태아일 위험성이 높다고 나오면, 확진을 위해 진단검사를 시행해서 확인해야 합니다.
- 지금 설명하고 있는 임신부의 혈액과 초음파 검사를 이용한 다운증후군 선별검사를 원하지 않으시면, 받지 않으셔도 됩니다.

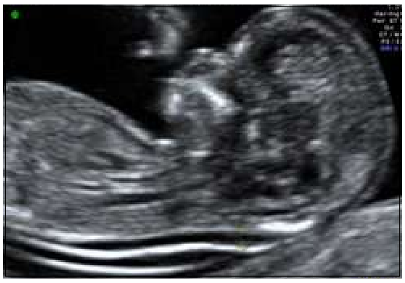

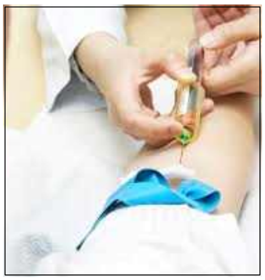

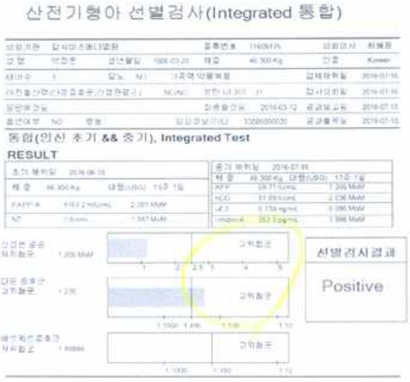

7. 지금 설명하고 있는 '**임신부의 혈액과 초음파 검사를 이용한 다운증후군 선별검사**'를 받을지 말지를 언제 결정하시겠습니까? (하나만 선택하세요)

① 지금 바로

② 며칠 동안 생각해 보고

③ 잘 모르겠음

8. 지금 설명하고 있는 '**임신부의 혈액과 초음파 검사를 이용한 다운증후군 선별검사**'를 받으시겠습니까? (하나만 선택하세요)

① 반드시 받겠음 ☞ 7페이지의 9번 질문으로 넘어가세요.

② 받는 것이 좋을 것 같음 ☞ 7페이지의 9번 질문으로 넘어가세요.

③ 받지 않는 것이 좋을 것 같음 ☞ 8페이지의 10번 질문으로 넘어가세요.

④ 절대 받지 않겠음 ☞ 8페이지의 10번 질문으로 넘어가세요.

9. 8번 문항에서 **받겠다고 선택하신 이유**로 가장 가까운 것을 고르세요. (**최대 세 개까지**만 선택하세요)

아기가 다운증후군이라 하더라도, 출산 전에 준비하고 계획해서 아기를 낳아서 키울 생각이 있어서	1
현실적으로 다운증후군 아기를 키우는 것이 불가능하기 때문에, 임신을 유지할지 종결할지를 결정하는데 도움이 될 수 있다고 생각해서	2
태아에 대해서 최대한 많은 정보를 알고 싶어서	3
태아에게 위험성이 전혀 없는 검사여서	4
배우자 또는 가족들이 내가 검사하기를 원해서	5
다른 임신부들이 모두 검사를 받아서	6
산부인과 의사가 권해서	7
임신 중 받을 수 있는 모든 검사는 다 받아야 할 것 같아서	8
기타: ______________________________	

☞ 이제 9페이지의 '시나리오 2'로 넘어가세요.

10. 8번 문항에서 **<u>받지 않겠다고 선택하신 이유</u>**로 가장 가까운 것을 고르세요. (**<u>최대 세 개까지</u>**만 선택하세요)

검사 결과에 상관없이 아기를 낳을 것이어서	1
본인이나 배우자 및 가족들이 다운증후군 아기를 키울 수 있다고 생각해서	2
아기에게 위험할 가능성이 있는 침습적인 검사는 시행하고 싶지 않아서	3
내 아기가 다운증후군의 고위험이라고 결과가 나올까봐 매우 걱정스럽고 불안해서, 차라리 안하는 것이 나을 것 같아서	4
분만하기 전까지 태아에 대한 정보를 너무 많이 알고 싶지 않아서	5
배우자 또는 가족들이 원치 않아서	6
내가 원치 않아서	7
이 검사는 최종진단을 알려주는 것이 아니어서	8
검사비가 경제적으로 부담이 되어서	9
검사를 시행하여 다운증후군을 발견한다 하더라도 현행 의료법상 다운증후군 태아를 유산을 하는 것은 불법이어서	10
기타: ______________________________	

☞ 이제 13페이지의 '시나리오 3'으로 바로 넘어가세요.

시나리오 2: 다운증후군 진단검사

당신은 임신부 혈액과 초음파 검사를 이용한 다운증후군 선별검사를 받기로 결정하고, 검사를 시행하였는데, 검사 결과가 다운증후군의 고위험군으로 판정되었습니다. 산부인과 의사로부터 **다운증후군 진단검사(융모막 검사, 양수검사, 제대혈 검사 등)**에 대해 다음과 같은 상담을 받았습니다.

- 지금 설명하고 있는 검사는 **태아가 다운증후군인지 아닌지를 확실히 알려주는 최종적인 진단**검사이며, **다운증후군 외의 태아 염색체의 구조적 이상과 수적 이상 여부** 또한 정확히 확인하는 진단검사입니다.
- 태아의 세포를 이용하기 때문에 **정확도가 가장 높은** 검사입니다.
- 이 검사는 초음파를 이용하여 임신부의 배에 주사바늘을 꽂아서 소량의 융모막검체나 양수검체, 또는 제대혈 검체를 채취하는 것으로 **약 0.1-0.3%의 유산이나 사산의 위험성**이 있는 것으로 알려져 있습니다.
- 지금 설명하고 있는 진단검사를 원하지 않으시면, 받지 않으셔도 됩니다.

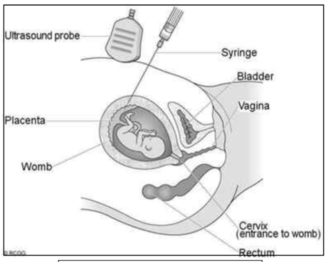

융모막검사

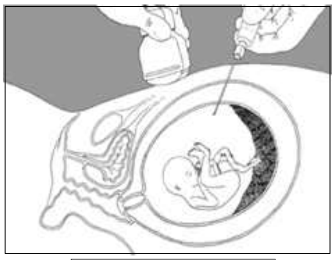

양수검사

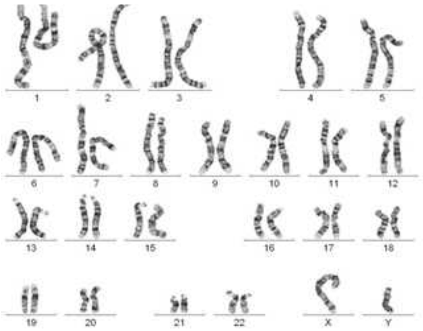

11. 지금 설명하고 있는 진단검사를 받을지 말지를 언제 결정하시겠습니까? (하나만 선택하세요)

① 지금 바로

② 며칠 동안 생각해 보고

③ 잘 모르겠음

12. 지금 시행하고자 하는 진단검사를 받으시겠습니까? (하나만 선택하세요)

① 반드시 받겠음 ☞ 11페이지의 13번 질문으로 넘어가세요.

② 받는 것이 좋을 것 같음 ☞ 11페이지의 13번 질문으로 넘어가세요.

③ 받지 않는 것이 좋을 것 같음 ☞ 12페이지의 14번 질문으로 넘어가세요.

④ 절대 받지 않겠음 ☞ 12페이지의 14번 질문으로 넘어가세요.

13. 12번 문항에서 **받겠다고 선택하신 이유**로 가장 가까운 것을 고르세요. (**최대 세 개**까지만 선택하세요)

아기가 다운증후군이라 하더라도, 출산 전에 준비하고 계획해서 아기를 낳아서 키울 생각이 있어서	1
현실적으로 다운증후군 아기를 키우는 것이 불가능하기 때문에, 임신을 유지할지 종결할지를 결정하는데 도움이 될 수 있다고 생각해서	2
태아에 대해서 최대한 많은 정보를 알고 싶어서	3
친구들이나 가족들 중에 임신 중에 이러한 진단검사를 시행받았던 경험이 있어서	4
배우자 또는 가족들이 내가 검사하기를 원해서	5
다른 임신부들이 모두 검사를 받아서	6
산부인과 의사가 권해서	7
임신 중 받을 수 있는 모든 검사는 다 받아야 할 것 같아서	8
기타: ______________________________	

☞ 이제 13페이지의 ‘시나리오 3’으로 넘어가세요.

14. 12번 문항에서 **<u>받지 않겠다고 선택하신 이유</u>**로 가장 가까운 것을 고르세요. (**<u>최대 세 개까지</u>**만 선택하세요)

유산이나 사산의 위험성이 있으므로	1
검사가 아프고 힘들 것 같아서	2
검사 결과에 상관없이 아기를 낳을 것이어서	3
본인이나 배우자 및 가족들이 다운증후군 아기를 키울 수 있다고 생각해서	4
내 아기가 다운증후군으로 진단될까봐 매우 걱정스럽고 불안해서, 차라리 안하는 것이 나을 것 같아서	5
임신을 유지해야 할지, 종결해야 할지를 결정해야 하는 상황을 원치 않아서	6
분만하기 전까지 태아에 대한 정보를 너무 많이 알고 싶지 않아서	7
배우자 또는 가족들이 원치 않아서	8
내가 원치 않아서	9
검사비가 경제적으로 부담이 되어서	10
검사를 시행하여 다운증후군을 발견한다 하더라도 현행 의료법상 다운증후군 태아를 유산을 하는 것은 불법이어서	11
기타: ______________________________	

☞ 이제 13페이지의 '시나리오 3'으로 넘어가세요.

시나리오 3: 임신부의 혈액내에 존재하는 태아 DNA를 이용한 다운증후군 선별검사(태아 DNA 검사)

최근 다운증후군 발견을 위한 새로운 유형의 검사가 개발되었는데, 이를 '**태아DNA를 이용한 다운증후군 선별검사(태아 DNA 검사)**'라고 합니다. 임신부의 혈액을 이용하기 때문에, 융모막검사, 양수검사 같은 침습적인 진단검사와 비교하여 비침습적 산전검사(Non-invasive prenatal testing, NIPT)라고도 불리기도 하지만, '태아 DNA 검사'라는 용어가 더 적절하여 본 설문지에서는 '태아 DNA 검사'라는 용어를 사용하겠습니다.

- 임신부의 혈액 속에는 태아의 세포와 DNA가 섞여 있는데, 태아 DNA 검사는 **임신부의 혈액에 있는 태아의 DNA를 이용**하여 태아가 다운증후군인지 아닌지를 선별하는 검사입니다.
- 임신부의 혈액을 이용하기 때문에, **유산이나 사산의 위험성이 증가하지 않습니다**.
- **다운증후군 발견율은 98% 이상으로** 알려져 있으며, 임신 10주경부터 시행할 수 있습니다.
- **태아 DNA 검사는 다운증후군을 최종 진단하는 검사는 아니므로**, 설령 태아 DNA 검사에서 태아가 다운증후군의 고위험군이라는 결과가 나왔다 하더라도, **반드시 확진을 위한 침습적인 진단검사를 시행해서 확인해야** 합니다.
- 초기에는 국내에서는 시행하는 기관이 없어서 임신부의 혈액을 해외로 보내서 검사를 진행하기도 하였으나, 최근 국내에서도 여러 회사에서 자체 개발하여 시행하고 있습니다.
- 이 검사는 다운증후군을 확진하는 검사는 아니지만, 98% 이상의 다운증후군 발견율을 보이며, 유산이나 사산의 위험이 없는 비침습적인 검사라는 점에서, 기존의 다운증후군 선별검사에서 고위험군으로 판정된 임신부에게 시행되거나, 기존의 다운증후군 선별검사 대신에 시행될 수 있는 선별검사입니다.

15. 태아 DNA 검사가 임신중 산전 관리의 일환으로서 실제 임신부에게 유용하게 사용될 것이라고 생각하십니까? (**하나**만 선택하세요)

① 확실히 그럴 것이다

② 아마 그럴 것이다

③ 아마 그렇지 않을 것이다

④ 확실히 그렇지 않을 것이다

16. 태아 DNA 검사가 임신중 산전 관리의 일환으로서 사용된다면, 본인은 태아 DNA 검사를 받을 의향이 있습니까? (**하나**만 선택하세요)

① 확실히 그럴 것이다

② 아마 그럴 것이다

③ 아마 그렇지 않을 것이다

④ 확실히 그렇지 않을 것이다

17. 이론적으로 태아 DNA 검사는 어떤 임신부가 받아야 한다고 생각하십니까? (**하나**만 선택하세요)

모든 임신부가 받아야 함	1
기존의 다운증후군 선별검사에서 고위험군으로 판정된 임신부가 받아야 함	2
기존의 다운증후군 선별검사에서 고위험군으로 판정된 임신부뿐만 아니라, 태아 DNA 검사를 원하는 모든 임신부는 모두 받아야 함<br>- 저위험군으로 판정되었으나 태아의 상태에 대한 불안이 심해서 태아 DNA 검사를 원하는 경우<br>- 기존의 다운증후군 선별검사를 시행하지 않고 바로 태아 DNA 검사를 원하는 경우 등	3
어떤 임신부도 받아서는 안 됨	4
기타: ______________________	

18. 다운증후군을 선별하기 위한 **<u>태아 DNA 검사를 받을지 말지 결정하는데 있어서 가장 중요한 요인</u>**을 하나만 고른다면 어떤 요인이라고 생각하십니까? (**<u>하나</u>**만 선택하세요)

① 태아에게 안전한 검사라는 점(유산이나 사산의 위험이 증가하지 않음)

② 다른 선별검사와 비교시 비교적 임신 초기에 다운증후군 고위험 여부를 알 수 있다는 점

③ 결과가 정확하다는 점(98%의 발견율)

④ 검사가 간편하다는 점(산모의 혈액만 채취하면 됨)

⑤ 나는 다운증후군 발견을 위한 산전 검사를 전혀 받지 않겠다.

⑥ 기타: ____

☞ 이제 16페이지의 ‘시나리오 4’로 넘어가세요.

시나리오 4: 임신부의 혈액내에 존재하는 태아 DNA를 이용한 다운증후군 선별검사(태아 DNA 검사)

최근에 당신은 임신을 확인하였습니다. 당신은 다운증후군 선별 검사를 받기로 결정하고, 검사를 시행하였는데, 검사 결과가 다운증후군의 고위험군으로 판정되었습니다. 산부인과 의사로부터 **임신부의 혈액내에 존재하는 태아 DNA를 이용한 다운증후군 선별검사(태아 DNA 검사)**에 대해 다음과 같은 상담을 받았습니다.

- 태아 DNA 검사는 태아가 다운증후군인지 아닌지를 선별하는 검사이며, **발견율은 98% 이상**입니다.
- 태아 DNA 검사는 산모의 팔에서 혈액을 채취하여 시행하는 검사로 **유산이나 사산의 위험성이 전혀 증가하지 않습니다**.
- 태아 DNA 검사는 다운증후군을 최종 진단하는 검사는 아니므로, 설령 태아 DNA 검사에서 태아가 다운증후군이라는 결과가 나왔다 하더라도, **반드시 확진을 위한 침습적인 진단 검사를 시행해서 확인해야** 합니다.
- 태아 DNA 검사는 **가격이 비싼 편**입니다. 50만원~100만원입니다.
- 태아 DNA 검사를 원하지 않으시면, 받지 않아도 됩니다.

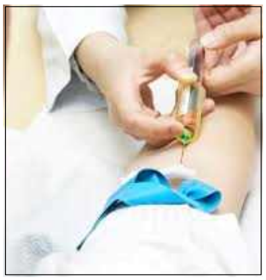

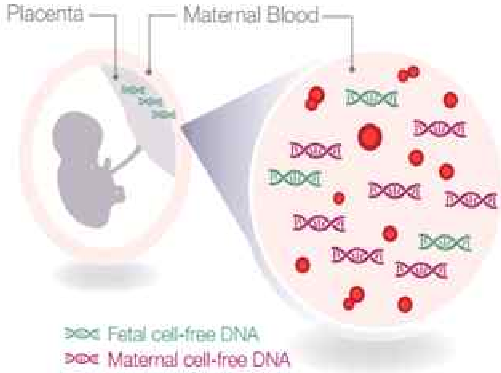

19. 지금 설명하고 있는 태아 DNA 검사를 받을지 말지를 언제 결정하시겠습니까? (하나만 선택하세요)

① 지금 바로

② 며칠 동안 생각해 보고

③ 잘 모르겠음

20. 지금 설명하고 있는 태아 DNA 검사를 받으시겠습니까? (하나만 선택하세요)

① 반드시 받겠음 ☞ 18페이지의 21번 질문으로 넘어가세요.

② 받는 것이 좋을 것 같음 ☞ 18페이지의 21번 질문으로 넘어가세요.

③ 받지 않는 것이 좋을 것 같음 ☞ 20페이지의 24번 질문으로 넘어가세요.

④ 절대 받지 않겠음 ☞ 20페이지의 24번 질문으로 넘어가세요.

21. 20번 문항에서 **받겠다고 선택하신 이유**로 가장 가까운 것을 고르세요. (**최대 세 개까지**만 선택하세요)

아기가 다운증후군이라 하더라도, 출산 전에 준비하고 계획해서 아기를 낳아서 키울 생각이 있어서	1
현실적으로 다운증후군 아기를 키우는 것이 불가능하기 때문에, 임신을 유지할지 종결할지를 결정하는데 도움이 될 수 있다고 생각해서	2
태아에 대해서 최대한 많은 정보를 알고 싶어서	3
태아에게 위험성이 전혀 없는 검사여서	4
배우자 또는 가족들이 내가 검사하기를 원해서	5
다른 임신부들도 많이 검사를 받는 것 같아서, 또는 내 친구들이나 가족들 중에 임신 중에 이러한 태아 DNA 검사를 시행 받았던 경험이 있어서	6
산부인과 의사가 권해서	7
임신 중 받을 수 있는 모든 검사는 다 받아야 할 것 같아서	8
기타: ________________	

22. 태아 DNA 검사에서 태아가 염색체이상이라는 결과가 나온다면 임신을 종결하시겠습니까? (**하나**만 선택하세요)

① 바로 종결하겠음

② 확진을 위한 진단검사를 받은 후 결과에 따라 결정하겠음

③ 잘 모르겠음

23. 태아 DNA 검사가 얼마 정도이면, 태아 DNA 검사를 받으실 의향이 있으신가요? (**하나**만 선택하세요)

① 5만원-10만원

② 10만원-20만원

③ 20만원-40만원

④ 40만원-60만원

⑤ 60만원-80만원

⑥ 80만원-100만원

⑦ 100만원 이상

⑧ 기타: ______________________________

☞ 수고하셨습니다. 21페이지의 설문지 작성자 본인에 대한 문답 부분으로 넘어가세요.

24. 20번 문항에서 **받지 않겠다고 선택하신 이유**로 가장 가까운 것을 고르세요. (**최대 세 개까지**만 선택하세요)

검사 결과에 상관없이 아기를 낳을 것이어서	1
본인이나 배우자 및 가족들이 다운증후군 아기를 키울 수 있다고 생각해서	2
내 아기가 다운증후군의 고위험이라는 결과가 나올까봐 매우 걱정스럽고 불안해서, 차라리 안하는 것이 나을 것 같아서	3
분만하기 전까지 태아에 대한 정보를 너무 많이 알고 싶지 않아서	4
배우자 또는 가족들이 원치 않아서	5
내가 원치 않아서	6
이 검사는 최종진단을 알려주는 것이 아니어서	7
검사비가 경제적으로 부담이 되어서	8
검사를 시행하여 다운증후군을 발견한다 하더라도 현행 의료법상 다운증후군 태아를 유산을 하는 것은 불법이어서	9
기타: ______________________________	

☞ 수고하셨습니다. 21페이지의 설문지 작성자 본인에 대한 문답 부분으로 넘어가세요.

<설문지 작성자 정보>

25. 당신의 성별은 무엇입니까?

① 남성

② 여성

26. 당신의 나이는 어떠한가요?   만 ___ 세

27. 당신의 최종학력은 무엇입니까?

① 교육받은 적이 없음

② 초등학교 졸업

③ 중학교 졸업

④ 고등학교 졸업

⑤ 대학교 졸업 또는 그 이상

⑥ 기타: ____________________

28. 당신은 종교를 가지고 있습니까?

① 없음

② 가톨릭교(천주교)

③ 기독교(개신교)

④ 불교

⑤ 이슬람교

⑥ 기타: ____________________

29. 당신은 현재 결혼을 하신 상태입니까?

① 예

② 아니오

30. 당신은 현재 자녀가 있습니까? (현재 임신은 제외합니다)

① 예

② 아니오

31. 당신은 현재 임신 몇 주입니까?   ___ 주

32. 당신은 현재 임신 또는 과거의 임신에서 임신부의 혈액과 초음파 검사를 이용한 다운증후군 선별검사를 시행 받은 적이 있습니까?

① 예

② 아니오

③ 잘 모르겠음

32-1 시행 받았다면, 그 결과는 어떻게 나왔습니까?

① 고위험군

② 저위험군

③ 잘 모르겠음

33. 당신은 현재 임신 또는 과거의 임신에서 다운증후군 진단검사를 시행 받은 적이 있습니까?

① 예

② 아니오

③ 잘 모르겠음

33-1 시행 받았다면, 그 결과는 어떻게 나왔습니까?

① 다운증후군으로 진단받음

② 다운증후군 외의 다른 염색체 이상으로 진단받음

③ 정상

④ 잘 모르겠음

34. 당신은 다운증후군 자녀를 두고 있습니까?

① 예

② 아니오

35. 당신은 다운증후군 자녀를 두고 있는 주위의 다른 사람(가족, 친척, 친구 포함)을 알고 있습니까?

① 예

② 아니오

<다음 문항은 2페이지 2번 문항에서 다운증후군 검사를 받을 의향이 없다고 표시하셨던 분들만 답해 주시면 됩니다.>

36. 본 설문지를 통해 임신 중 다운증후군 검사에 대해 알아보았습니다. 향후 다운증후군 검사를 받을 의향이 있나요?

① 예

② 아니오

37. 다운증후군 검사는 앞서 설명드린대로 ① 선별검사와 진단검사를 단계적으로 시행하는 방법과, ② 선별검사를 시행하지 않고 곧바로 진단검사를 시행하는 방법이 있습니다. 어떤 검사 방법을 선택하시겠습니까?

① 선별검사와 진단검사를 단계적으로 시행

② 선별검사 없이 곧바로 진단검사를 시행

③ 어떠한 다운증후군 검사도 받지 않겠다

38. **태아 DNA 검사**가 임신중 산전 관리의 일환으로서 사용된다면, 본인은 태아 DNA 검사를 받을 의향이 있습니까? (**하나**만 선택하세요)

① 확실히 그럴 것이다

② 아마 그럴 것이다

③ 아마 그렇지 않을 것이다

④ 확실히 그렇지 않을 것이다

⑤ 어떠한 다운증후군 검사도 받지 않겠다

<설문에 응해 주셔서 대단히 감사합니다.>
