# Supplementary material for: Prospective observations study protocol to investigate cost-effectiveness of various prenatal test strategies after the introduction of noninvasive prenatal testing
Source: BMC Pregnancy Childbirth. 2018 Jul 24;18:307. doi: 10.1186/s12884-018-1930-y (PMC6056912; doi:10.1186/s12884-018-1930-y)
Supplement: Supplementary file 2 — Physicians Questionnaire: Korean version and Physicians Questionnaire: English version. (ZIP 536 kb) [file 12884_2018_1930_MOESM2_ESM.zip › (Additional file 2) physicians questionnaire_korean versionR2.pdf]

NIPT 의료진 설문지

설문의 목적 : 이 설문은 산전 진단에 대하여 현재 시행하고 있는 산부인과 전문의 선생님들의 의견 및 경향성을 알아보고자 합니다. 또한 이 설문은 NIPT(비침습적 산전검사)의 도입에 따른 새로운 진료 지침 개발에 반영할 수 있습니다.

1. 선생님은 다음 중 어떠한 진료 환경에서 근무하고 계십니까?
   (1) 대학병원
   (2) 준종합병원
   (3) 분만을 하는 개원병원
   (4) 분만을 하지 않는 개원병원
   (5) 기타 ____

2. 임상진료를 하신 지 얼마나 되셨나요?
   (1) 5년 이하
   (2) 6년 ~ 10년
   (3) 11년 ~ 15년
   (4) 16년 ~ 20년
   (5) 21년 이상

3. 전임의를 수료하셨나요? 수료하셨다면 어떤 분야에 해당하나요?

______ 예

______ 아니오

3-1. '예'라고 답하셨다면 → 전공분야를 선택해 주세요

________ 산과

________ 내분비

________ 부인과

________ 기타 (구체적으로)__________________________________

4. 한 달에 진료하는 외래 산모 수는? ____________________ 명

5. 분만을 하신다면 한 달 평균 분만 산모 수는? ____________________ 명

6. 선생님의 성별은?

_____ 남

_____ 여

7. 선생님의 나이는? 만 _______________ 세

8. 선생님은 태아 염색체 이상을 위한 검사를 상담할 때, 고위험산모와 저위험산모를 구분하여 산전검사를 권유하시나요?
_____ 예
_____ 아니오

8-1 만약 '예'라고 답하셨다면, 다음 중 어떤 항목에 따라 산모를 고위험으로 분류하나요? (중복가능하며, 해당하는 모든 항목을 체크해주세요)
____ 산모의 나이
____ 산모혈청검사 고위험 결과
____ 태아 염색체 이상을 의심할 만한 초음파 소견
____ 부모의 염색체 이상
____ 이전에 염색체 이상의 태아를 임신한 과거력
____ 기타 (구체적으로 적어주세요)
____
____

9. 선생님은 **Aneuploidy testing** (serum screening and/or invasive diagnostic testing)을 **모든 산모에게** 권해야 한다고 생각하나요? 아니면 **고위험 산모에게만** 권해야 한다고 생각하나요?
____ 모든 임산부에게 권해야 한다
____ 고위험 임산부에게만 권해야 한다

10. **혈청 선별검사의** 장점과 단점에 대하여 각각 항목별로 중요성의 정도에 따라서 **점수**를 표시해 주세요. (1~5 점 중 전혀 동의하지 않거나 전혀 중요하지 않다고 생각하면 1 점, 제일 중요하거나 전적으로 동의한다면 5 점)

**혈청 선별검사의 장점**	**동의하거나 중요하다고 생각하는 정도(1~5 점)**
값이 싸다	
환자들이 잘 이해하고 받아들인다	
보험적용이 된다	
검사에 대해 축적된 경험과 데이터가 많다	
높은 detection rate	
비침습적이다	
기존의 지침에 따라 권유되는 검사이다	

**혈청 선별검사의 단점**	**동의하거나 중요하다고 생각하는 정도(1~5 점)**
높은 위양성	
NT 초음파와 불일치(inconsistency)	
위양성과 관련한 환자의 불안감	
이어지는 침습적 검사의 위험도	

11. 선생님께서 NIPT 검사를 처방하는데 장점과 단점에 대하여 각각 항목별로 중요성의 정도에 따라서 **점수**를 표시해 주세요. (1~5 점 중 전혀 동의하지 않거나 전혀 중요하지 않다고 생각하면 1 점, 제일 중요하거나 전적으로 동의한다면 5 점)

**NIPT 검사의 장점**	**동의하거나 중요하다고 생각하는 정도(1~5 점)**
이른 임신 주수부터 검사가 가능하다	
환자들이 잘 이해하고 받아들인다	
침습적 검사보다 비용이 싸다	
높은 detection rate	
비침습적이다	
위양성률이 낮다	

**NIPT 검사의 단점**	**동의하거나 중요하다고 생각하는 정도(1~5 점)**
비용이 비싸다	
고위험군일 경우 침습적 확진검사가 필요하다	
환자들의 검사에 대한 이해도가 부족하다	
결과가 제한적이다<br>(침습적 검사에 비하여 제한적인 염색체 이상에 대한 위험도 결과를 알 수 있다.)	

기타 의견이 있다면 구체적으로 기술해 주세요

____

____

12. 선생님이 **단태아 산모**에게 시행하고 있는 산모혈청 검사는 무엇인가요?

_____ Triple test
_____ Quadruple test
_____ Combined test
_____ Integrated test
_____ Sequential test

13. 선생님이 **쌍태아 산모**에게 시행하고 있는 산모혈청 검사는 무엇인가요?

_____ Triple test
_____ Quadruple test
_____ Combined test
_____ Integrated test
_____ Sequential test

14. 선생님은 한 달에 평균 몇 건의 **침습적 검사를 시행**(또는 의뢰)하나요?

**개수**	**침습적 검사**
	Amniocentesis
	Chorionic villous sampling
	기타____

15. 선생님의 산전 검사에 관한 진료의 standard flow 는 다음 중 어떤 항목에 영향을 받아 정해지나요? (가장 영향을 받는 한 가지를 고르세요)

	**진료의**에 의하여 환자 개별적으로 선택
	**진료의와 환자, 보호자와** 논의를 통한 비공식적 consensus 에 따라
	과별 또는 부서별의 방침에 따라
	병원 행정상 또는 병원 진료위원회의 방침에 따라
	산부인과 학회의 방침에 따라
	기타____

16. 산전검사 (prenatal screening) 에 대하여 주로 언제 설명하시나요?

______ 임신 후 첫 방문시

______ 8~10 주

______ 11-13 주

______ 16-18 주

______ 기타 (구체적으로 적어주세요)____

17. 일반적으로 산전검사에 대한 설명을 할 때 시간은 얼마나 걸리나요?

_____ 1 분 이하

_____ 1~3 분

_____ 4~5 분

_____ 5~7 분

_____ 8~10 분

_____ 10 분 이상 (몇 분인지 적어주세요) ____

18. 산전검사 결과에서 **양성소견이 있을 경우** 상담을 할 때 시간은 얼마나 걸리나요?

_____ 1 분 이하

_____ 1~3 분

_____ 4~5 분

_____ 5~7 분

_____ 8~10 분

_____ 10 분 이상 (몇 분인지 적어주세요) ____

19. 귀 병원에서는 유전상담(genetic counseling)을 누가 담당하나요?

________ 산과의

________ 유전상담을 전문하는 특정 산과의

________ 소아과의

________ 유전상담사

________ 기타 (구체적으로 적어주세요) ____

20. NIPT 또는 산전검사에 대한 자체 설명문 또는 브로슈어 같은 것이 따로 있나요?

______ 예

______ 아니오

21. 산전 검사를 할 때 동의서를 받고 시행하나요?

______ 예

______ 아니오

22. 현재 임상 진료를 할 때 NIPT 를 사용하고 계시나요?

____ 예

____ 아니오

22-1.'**아니오'를 선택한 경우, 그 이유는 다음 중 무엇인가요?**

____ NIPT 검사의 유용성과 필요성을 인지하고 있지만, 아직 병원에 처방코드가 없다

____ NIPT 에 대하여 잘 알고 있지만, 아직 처방을 해 본 적이 없다

____ NIPT 에 대하여 잘 알고 있지만, 필요 없다고 생각한다

____ NIPT 에 대하여 논문을 보거나 발표를 들은 적은 있지만, 아직 익숙하지 않다

____ NIPT 에 대하여 들어본 적이 없다

____ 기타 (구체적으로 적어주세요)____

22-2. **'예'를 선택한 경우,** 다음 중 어떤 경우에 NIPT 검사를 고려하나요? **(복수선택 가능)**

____ 환자가 원할 때

____ 고령 임산부일 경우

____ 이전 염색체 이상의 태아를 임신한 과거력이 있는 경우

____ 부모 염색체 이상 또는 염색체 이상의 가족력이 있을 경우

____ 산모혈청검사에서 고위험 결과를 보일 때

____ 1 삼분기 초음파에서 이상을 보일 때 (NT 초음파)

22-3. 귀 병원에서 사용하고 있는 NIPT 의 종류는?

______ Sequenom MaterniT21™

______ Verinata Verifi™

______ Ariosa Harmony™

______ Natera Panorama™

______ 녹십자 G-NIPT™

______ BGI 니프티(NIPTY™)

______ 마크로젠 faest™

______ 랩지노믹스 MomGuard™

______ 기타 (구체적으로 적어주세요)____

23. 여러 회사의 NIPT 중 한 가지를 선택하는 기준은? **(복수선택 가능)**

	**비용**
	**진료의의 개인적인 선호도**
	검사별 결과 수치에 따라 (**sensitivity, specificity 등**)
	**과별 또는 부서별 방침**에 따라
	**병원** 행정적 또는 진료위원회의 방침에 따라
	기타________________________________

24. 선생님이 생각하기에 선생님 환자들의 NIPT 에 대한 관심은 어느 정도라고 생각하십니까?

____ 관심이 있다

____ 중립적이다

____ 관심이 없다

____ 잘 모른다

25. 태아 염색체 이상에 대한 **고위험 산모에게 NIPT 를 권할** 때 다음 중 각각의 임상 상황에 대하여 NIPT 권유에 대하여 어떻게 생각하시나요?

	Agree	Unsure	Disagree
고위험 산모에게 일차적 산전검사로 (단일검사로)			
고위험 산모에게 초음파와 더불어 일차적 산전검사로			
이차적 검사로 (초음파 이상소견이 있거나, 혈청검사 고위험일 때)			

26. 태아 염색체 이상에 대한 **중등도 위험(average-risk) 산모에게 NIPT 를 권할** 때 다음 중 각각의 임상 상황에 대하여 NIPT 권유에 대하여 어떻게 생각하시나요?

	Agree	Unsure	Disagree
모든 산모에게 일차적 산전검사로 (단일검사로)			
모든 산모에게 초음파와 더불어 일차적 산전검사로			
이차적 검사로 (초음파 이상소견이 있거나, 혈청검사 고위험일 때)			

27. NIPT 에 대하여 환자 및 보호자에게 설명 할 때, 발견율(detection rate)을 얼마라고 설명하시나요?

_______ 100%

_______ 99%

_______ 98%

_______ 97%

_______ 96%

_______ 기타 (구체적으로 적어주세요) ______________________________ %

28. NIPT 검사에서 고위험이 나왔다면, 태아가 실제로 염색체 이상이 나올 확률은 몇 퍼센트라고 설명하시나요? **(주관식)**

______________________ %

29. NIPT 검사에서 고위험이 나왔다면, 다음으로 하시는 것은? **(복수선택 가능)**

______ CVS

______ Amniocentesis

______ 타병원 전원

______ 임신종결

______ 기타 (구체적으로 적어주세요) ______________________________

30. NIPT 에서 'No call' 결과가 나왔다면 다음으로 하시는 것은?

______ 아직까지 그런 적이 없어서 잘 모르겠다

______ maternal serum test

______ CVS

______ Amniocentesis

______ repeat NIPT

______ 기타 (구체적으로 적어주세요) ______________________________

31. NIPT 를 **시행하기 전 상담 내용에** 관하여 알고 있는 항목을 선택해 주세요.

	산모혈청검사와 비교할 때 발견율이 높다는 장점이 있다
	위음성률이 낮아서 고위험으로 나오는 결과가 적어지므로 invasive test 건수를 줄일 수 있다
	다운증후군에 대한 음성예측값이 높아서 invasive test 로 인한 유산의 위험을 걱정하는 환자들에게 중요한 의미가 있다
	임신 주수에 따라 위험도 계산이 달라지지 않는다<br>(염색체 이상에 대한 위험도가 임신 주수에 덜 의존적이다)
	NIPT 가 진단검사는 아니지만 매우 높은 민감도와 특이도를 가지고 있다
	NIPT 검사에서 저위험군으로 결과가 나오더라도 태아 염색체 이상에 대한 2%의 잠재적 위험도가 존재한다

	검체에 따라 분석이 불가능한 경우가 있다
	NIPT 검사를 시행하더라도 임신 15 – 20 주 사이에 이루어지는 신경관 결손 선별검사인 모체혈청 AFP 검사는 별도로 받아야 한다
	피험자 동의서(informed consent)를 받아야 한다

32. NIPT 를 시행한 뒤 **결과 상담 시에** 포함되어야 할 내용에 관하여 알고 있는 항목을 선택해 주세요

	매우 낮지만 위음성 가능성이 있으므로 저위험군의 결과가 나왔더라도 다운증후군일 가능성이 있다
	위양성의 가능성이 있으므로 고위험군의 결과가 나올 경우, 반드시 적절한 유전상담과 함께 융모막검사나 양수검사를 통한 확진 검사가 필요하다.
	태아 DNA 양이 적거나 (low fetal fraction) 또는 여러 가지 이유로 검사 결과가 보고되지 않는 경우에는 ("no call" test result) 태아의 염색체 이상의 위험이 증가된다는 보고가 있으므로 재검사 또는 융모막 검사나 양수 검사 등의 진단 검사 등에 대한 자세한 유전상담을 받아야 한다.
	저위험으로 나온 경우에도 residual risk 가 있음을 설명해야 한다

33. 선생님이 생각하기에 NIPT 의 가격이 어느 정도라면 중등도 위험군 환자에게 NIPT 를 일차적으로 권하시겠습니까? 가격을 적어주세요.

____________________ 만원

34. NIPT 로 성염색체에 대한 선별검사를 하는 것에 대하여 어떻게 생각하시나요?

______ 긍정적이다
______ 부정적이다
______ 모르겠다

35. NIPT 로 미세결실을 추가하여 검사하는 것에 대하여 어떻게 생각하시나요?

______ 긍정적이다
______ 부정적이다
______ 모르겠다

36. NIPT 에 관한 정보는 주로 어디에서 얻고 계신가요?

______ 논문 검색을 통하여
______ 학회 등의 발표를 통하여
______ 학회 등의 진료지침 발표안을 통하여
______ 책을 통하여
______ 동료들과 의견교환을 통하여
______ 기타 (구체적으로 적어주세요) ____

37. 산전 진단 관련하여 아쉽거나 필요한 것으로 생각되는 점이 있으면 적어주세요.

긴 시간 설문에 응해주셔서 매우 감사 드립니다. 선생님의 관심과 참여로 산부인과 진료지침 개발에 큰 도움이 될 수 있습니다. 본인 확인 및 중복설문을 피하기 위하여 선생님의 성함을 적어주세요. 수집된 개인정보는 타인에게 제공되지 아니하며, 제시된 목적을 위해서만 사용됨을 알려드립니다.

성함 : ______________________________

설문 날짜 : ________________________
