# Supplementary material for: Prospective observations study protocol to investigate cost-effectiveness of various prenatal test strategies after the introduction of noninvasive prenatal testing
Source: BMC Pregnancy Childbirth. 2018 Jul 24;18:307. doi: 10.1186/s12884-018-1930-y (PMC6056912; doi:10.1186/s12884-018-1930-y)
Supplement: Supplementary file 2 — Physicians Questionnaire: Korean version and Physicians Questionnaire: English version. (ZIP 536 kb) [file 12884_2018_1930_MOESM2_ESM.zip › (Additional file 2)physicians questionnaire_english versionR2.pdf]

Survey about physicians' attitudes about prenatal testing including non-invasive prenatal testing for aneuploidy

Purpose of survey: This survey asks board-certified obstetrician-gynecologists about their current practice patterns regarding prenatal testing for fetal aneuploidy.

Demographic information

1. Which describes the practice setting in which you see your patients?
   _____ Academic hospital
   _____ General hospital
   _____ Private hospital with delivery room
   _____ Private hospital without delivery room
   _____ Other (specify) _____________________

2. How many years have you practiced as an obstetrician-gynecologist?
   _____ < 5 years
   _____ 6 ~ 10 years
   _____ 11 ~ 15 years
   _____ 16 ~ 20 years
   _____ > 21 years

3. Did you complete a fellowship?
   _____ No
   _____ Yes (Obstetrics)
   _____ Yes (Endocrinology)
   _____ Yes (Gynecology)
   _____ Yes (Others) (specify) ____________________

4. How many new obstetrical patients do you see in a typical month? _____

5. What is the average number of deliveries in your practice in a typical month? _____

6. What is your gender?
   _____ male
   _____ female

7. What is your age? _____

General prenatal screening practices

8. Do you regularly identify a subset of your patients as being at high-risk for fetal aneuploidy?
   _____ Yes
   _____ No

   If yes, which of the following criteria do you use to determine that a patient is at high-risk for carrying an aneuploidy pregnancy? (check all that apply)

_____ Maternal age
_____ Positive screening results
_____ Ultrasound findings suggestive of aneuploidy
_____ Family history of aneuploidy
_____ Previous pregnancy affected by aneuploidy
_____ Others (specify) ________________________

9. Do you believe that aneuploidy testing (screening and/or invasive diagnostic testing) should be offered to all pregnant women or only high-risk pregnant women?
_____ Aneuploidy testing should be offered to all pregnant women
_____ Aneuploidy testing should be offered to only high-risk pregnant women

10. How much do you agree with each of the items listed below regarding **<u>conventional maternal serum screening tests</u>** on a scale of 1-5, **where 5 = completely agree and 1 = completely disagree**?

**Advantages of conventional screening tests**	**Agree that it is important (5-1)**<br>**5 = completely agree, 1 = completely disagree**
Inexpensive	
Well accepted by patients	
Covered by insurance	
Long history and experience with the test	
High detection rate (test accuracy)	
Safe for the fetus	
Recommended by professional societies	

**Disadvantages of conventional screening tests**	**Agree that it is important (5-1)**<br>**5 = completely agree, 1 = completely disagree**
High false positives	
Inconsistency between NT ultrasound and the test	
Patient anxiety related to false positive results	
Risks associated with follow-up invasive testing	

11. How much do you agree with each of the items listed below regarding **<u>Non-Invasive Prenatal Testing (NIPT)</u>** on a scale of 1-5, **where 5 = completely agree and 1 = completely disagree**?

**Advantages of NIPT**	**Agree that it is important (5-1)**<br>**5 = completely agree, 1 = completely disagree**
Available in the first trimester	
Well accepted by patients	
Less expensive than invasive test	
High detection rate	
Safe for the fetus	
Low false positives	

**Disadvantages of NIPT**	**Agree that it is important (5-1)**

	5 = completely agree, 1 = completely disagree
More expensive than conventional screening	
Should confirm results using invasive test	
Lack of knowledge of NIPT by patients	
Limited results (Commercialized NIPT tests only a few chromosomes)	

12. What test do you currently provide for detection of aneuploidy in singletone pregnancies? (check all that apply)
_____ Triple test
_____ Quadruple test
_____ Combined test
_____ Integrated test
_____ Sequential test

13. What test do you currently provide for detection of aneuploidy in multiple pregnancies? (check all that apply)
_____ Triple test
_____ Quadruple test
_____ Combined test
_____ Integrated test
_____ Sequential test

14. How many invasive prenatal testing procedures per month do you perform or refer to another specialist to perform?

**Invasive procedure type**	**Average number of procedures performed per month**
Amniocentesis	
Chorionic villous sampling	
Others	

15. Which statement below describes how standard practice is established within your office or institution, particularly regarding the adoption of new technologies? (Choose the best answer below)

	Individual choice by physician
	Informal consensus among physicians based on group discussions
	Department chairman, medical director (or comparable individual) sets the policy
	Hospital P&T committee or other administrative body sets the policy
	Policy adjusts automatically according to society guidelines
	Other (specify)

16. When do you usually inform your patients about prenatal tests for aneuploidy?
_____ the first visit
_____ 8~10 weeks of gestation
_____ 11~13 weeks of gestation
_____ 16~18 weeks of gestation
_____ Other (specify)

17. How long does it take you to inform your patients about prenatal tests for aneuploidy?
_____ < 1 minute
_____ 1~3 minutes
_____ 4~5 minutes
_____ 5~7 minutes
_____ 8~10 minutes
_____ > 10 minutes (specify) ________

18. If there are positive test results from prenatal screening, how long does it take for you to counsel patients?
_____ < 1 minute
_____ 1~3 minutes
_____ 4~5 minutes
_____ 5~7 minutes
_____ 8~10 minutes
_____ > 10 minutes (specify) ________

19. Who mainly provides detailed genetic counseling in your hospital?
_____ Obstetricians
_____ Certain obstetricians who specialize in genetic counseling
_____ Pediatricians
_____ Clinical geneticists
_____ Others (specify) ________

20. Do you get an informed consent before the prenatal test?
_____ Yes
_____ No

Questions regarding NIPT

21. Do you have your own pre-prepared explanatory notes or brochures about prenatal test or NIPT?
_____ Yes
_____ No

22. Do you have an experience using NIPT?
_____ Yes
_____ No

23. How would you rate your patients' level of interest in NIPT?
_____ Interested
_____ Neutral
_____ No interested
_____ They don't know NIPT

24. Do you generally get informed consent before NIPT?
_____ Yes
_____ No

25. How likely are you to adopt NIPT for testing patients **at high-risk for fetal trisomy** in each of the clinical situations described below? Select the best answer in each case.

	Agree	Unsure	Disagree
Primary screening in all high-risk patients (as sole test)			
Primary screening in all high-risk patients along with ultrasound			
Secondary screening<br>(abnormal ultrasound or positive serum screening result)			

26. How likely are you to adopt NIPT for testing patients **at average-risk for fetal trisomy** in each of the clinical situations described below? Select the best answer in each case.

	Agree	Unsure	Disagree
Primary screening in all high-risk patients (as sole test)			
Primary screening in all high-risk patients along with ultrasound			
Secondary screening<br>(abnormal ultrasound or positive serum screening result)			

27. What do you inform your patients about detection rate of NIPT?
_____ 100%
_____ 99%
_____ 98%
_____ 97%
_____ 96%
_____ Other (specify)

28. If the result of NIPT was high risk, what do you inform your patient about the positive predictive value of NIPT? (a chance that the fetus has true positive for fetal trisomy)
__________ %

29. What is the next step if the result of NIPT was high risk?
_____ CVS
_____ Amniocentesis
_____ transfer to tertiary hospital
_____ consider termination of pregnancy
_____ Other (specify)

30. What is your next step if the result of NIPT is "No call" (uninformative result) ?
_____ maternal serum test
_____ CVS
_____ amniocentesis
_____ repeat NIPT
_____ I don't know because I don't have any experience of "No call"
_____ Other (specify)

31. Please check all of the information that you already know about **the pretest counseling of NIPT**.

	Detection rates are higher than those of conventional maternal serum screening for traditionally screened aneuploidies
	NIPT has a lower false-positive rate, meaning fewer women will receive a “positive” screen, necessitating fewer invasive procedures
	There is a high negative predictive value for Down syndrome. This may be important for patients seeking to avoid the risks (e.g. fetal loss) inherent with invasive testing
	Risk assessment is less dependent on gestational age
	NIPT is not diagnostic but it is the most sensitive and specific screening option
	Even when the NIPT result is negative, there still remains a 2% potential risk of chromosomal abnormalities
	A “no-call” result may be reported (uninformative test result)
	NIPT does not screen for open neural tube defects. Maternal serum α-fetoprotein testing should still be offered at 15–20 weeks gestation to screen for open neural tube defects
	Informed consent is required prior to NIPT

32. Please check all of the information that you already know about post-test counseling of NIPT.

	There is a possibility of false-negative screening results
	There is a possibility of false-positive screening results, necessitating recommendation of confirmative testing (CVS or amniocentesis)
	Diagnostic testing should be offered for a “no-call” NIPT result
	When a screen-negative result is encountered, residual risk should be reinforced

33. At what price would you recommend NIPT to your patients at average-risk for fetal trisomy as a first-line screening test?
    ___________ won (Korean currency)

34. What is your opinion about the clinical relevance of <u>prenatal screening for sex chromosome</u>?
    _____ positive
    _____ negative
    _____ neutral

35. What is your opinion about the clinical relevance of <u>prenatal screening for microdeletions</u>?
    _____ positive
    _____ negative
    _____ neutral

36. How did you learn about NIPT? (check all that apply)
    _____ Searching for related articles
    _____ Educational lecture or conference
    _____ Professional society’s recommendations or guidelines
    _____ Books
    _____ Group discussion among colleagues
    _____ others (specify) _________________________
